# Biology in the Dry Seed: Transcriptome Changes Associated with Dry Seed Dormancy and Dormancy Loss in the *Arabidopsis* GA-Insensitive *sleepy1-2* Mutant

**DOI:** 10.3389/fpls.2017.02158

**Published:** 2017-12-22

**Authors:** Sven K. Nelson, Tohru Ariizumi, Camille M. Steber

**Affiliations:** ^1^Molecular Plant Sciences Program, Washington State University, Pullman, WA, United States; ^2^Department of Crop and Soil Science, Washington State University, Pullman, WA, United States; ^3^Wheat Health, Genetics, and Quality Research Unit, United States Department of Agriculture–Agricultural Research Service, Pullman, WA, United States

**Keywords:** SLY1, Arabidopsis, dormancy, dry after-ripening, germination, seeds, transcriptome, DELLA

## Abstract

Plant embryos can survive years in a desiccated, quiescent state within seeds. In many species, seeds are dormant and unable to germinate at maturity. They acquire the capacity to germinate through a period of dry storage called after-ripening (AR), a biological process that occurs at 5–15% moisture when most metabolic processes cease. Because stored transcripts are among the first proteins translated upon water uptake, they likely impact germination potential. Transcriptome changes associated with the increased seed dormancy of the GA-insensitive *sly1-2* mutant, and with dormancy loss through long *sly1-2* after-ripening (19 months) were characterized in dry seeds. The *SLY1* gene was needed for proper down-regulation of translation-associated genes in mature dry seeds, and for AR up-regulation of these genes in germinating seeds. Thus, *sly1-2* seed dormancy may result partly from failure to properly regulate protein translation, and partly from observed differences in transcription factor mRNA levels. Two positive regulators of seed dormancy, DELLA *GAI* (*GA-INSENSITIVE*) and the histone deacetylase *HDA6/SIL1* (*MODIFIERS OF SILENCING1*) were strongly AR-down-regulated. These transcriptional changes appeared to be functionally relevant since loss of *GAI* function and application of a histone deacetylase inhibitor led to decreased *sly1-2* seed dormancy. Thus, after-ripening may increase germination potential over time by reducing dormancy-promoting stored transcript levels. Differences in transcript accumulation with after-ripening correlated to differences in transcript stability, such that stable mRNAs appeared AR-up-regulated, and unstable transcripts AR-down-regulated. Thus, relative transcript levels may change with dry after-ripening partly as a consequence of differences in mRNA turnover.

## Introduction

Plant colonization of dry land was made possible by the evolution of seeds as a means of propagation. The plant embryo encapsulated in orthodox seeds can survive long periods in a desiccated, quiescent state, allowing time for dispersal (reviewed in Bewley et al., 2013). Osmoprotectants like LEA (Late Embryogenesis Abundant) proteins and non-reducing sugars protect desiccated seeds from cellular damage due to destabilization of membranes and proteins. Non-reducing sugars and compatible solutes replace water in dry seeds at 5–15% moisture, resulting in a “glassy state” that allows only gradual molecular movement (Buitink and Leprince, 2004). Ribosomes are inactive in dry seeds, but form polysomes without *de novo* translation during water uptake or imbibition (Spiegel and Marcus, 1975; Rajjou et al., 2004). mRNAs transcribed during seed maturation are stored in dry seeds, and likely play an important role in determining whether or not a seed can germinate because they encode the earliest proteins translated during seed germination (Marcus and Feeley, 1964; Dure and Waters, 1965; Waters and Dure, 1965, 1966; Chen et al., 1968; Gordon and Payne, 1976; Ishibashi et al., 1990; Almoguera and Jordano, 1992).

Seed dormancy is an adaptation that prevents seed germination even when immediate environmental conditions are favorable (Finch-Savage and Leubner-Metzger, 2006). Seed dormancy prevents germination out of season, allows time for seed dispersal, and increases the variation in the timing of germination (reviewed in Koornneef and Alonso-Blanco, 2000; Venable, 2007; Poisot et al., 2011). Seed dormancy is established during embryo maturation, the final stage of seed development. Dormancy can be relieved through a period of dry storage called after-ripening, through moist chilling (cold stratification), or through seed coat scarification. The after-ripening time required for dormancy loss depends on genotype, and can be perturbed through altered function of dormancy-regulating genes (Ariizumi and Steber, 2007; Chiang et al., 2011; Kendall et al., 2011; reviewed in Koornneef and Alonso-Blanco, 2000; reviewed in Nonogaki, 2014). This genetic variation is particularly important in cereal crops where lack of seed dormancy can lead to problems with preharvest sprouting, the germination of grain on the mother plant when cool and rainy conditions occur before harvest (reviewed by Rodríguez et al., 2015). Informed genetic strategies may allow us to increase seed dormancy sufficiently to prevent preharvest sprouting without causing problems with poor germination and emergence when winter crops are planted in the fall with little after-ripening.

The word “germination” refers both to a process and an event. The germination process has been divided into three phases (reviewed in Bewley et al., 2013). During Phase I, rapid water uptake (imbibition) leads to cellular rehydration associated with expression of genes involved in seed maturation and desiccation tolerance such as LEAs, small heat shock proteins (smHSPs) and oxidoreductases. During Phase II, water uptake plateaus and the seed undergoes essential processes, including DNA repair, initiation of transcription and translation, mitochondrial repair, respiration, initiation of stored nutrient mobilization, DNA synthesis, and cell expansion. Phase III begins with germination the event (germination *per se*), defined by embryonic root emergence. Phase III also includes post-germinative events such as completion of nutrient mobilization, cell division, and seedling growth. Living dormant seeds do not reach Phase III, but they do imbibe water and enter Phase II. This paper will refer to ungerminated seed in Phase I or II as “imbibing seeds” to distinguish them from seeds undergoing germination *per se*.

Understanding how dormancy loss through after-ripening occurs in a dry and metabolically quiescent seed is one of the great mysteries of plant science (reviewed in Koornneef and Alonso-Blanco, 2000; Bewley et al., 2013). Changes during dry seed storage regulate germination potential once the seed is imbibed, yet the severe water deficit in dry seeds likely inhibits most biological processes, including transcription and translation. Transcriptome studies have observed differential accumulation of stored dry seed mRNAs with after-ripening of multiple species (Comai and Harada, 1990; Bove et al., 2005; Leubner-Metzger, 2005; Cadman et al., 2006; Leymarie et al., 2007; Oracz et al., 2007; Bazin et al., 2011; Chitnis et al., 2014; Meimoun et al., 2014). The changes in transcript levels with dry seed after-ripening may result from transcription or differential mRNA turnover. Based on inhibitor studies, protein translation, but not gene transcription, is required for seed germination (Spiegel and Marcus, 1975; Rajjou et al., 2004). This emphasizes the importance of stored mRNAs, since translation of stored mRNA is necessary and sufficient for seed germination.

Some have hypothesized that localized moisture conditions may allow active transcription in dry seeds, while others maintain this is unlikely. Hydrogen proton NMR microimaging of dry seeds detected possible moisture pockets proposed to make dry seed transcription possible (Leubner-Metzger, 2005). Polysome profiles of nuclei isolated from dry seeds of *Brassica napus* suggested active transcription, albeit at 8% of the rate observed during seed maturation (Comai and Harada, 1990). However, non-transcriptional processes likely cause apparent changes in relative transcript abundances during dry seed after-ripening (reviewed in Bewley et al., 2013). Differential RNA turnover may be triggered by mRNA oxidation resulting from oxygen diffusion into dry seeds (Oracz et al., 2007). Dry seed after-ripening of sunflower (*Helianthus annuus*) was associated with differential transcript levels, including 24 after-ripening-down-regulated mRNAs preferentially targeted for destruction by mRNA oxidation (Bazin et al., 2011). Oxidative reactions have also been implicated in dormancy regulation through lipid peroxidation, carbonylation of specific proteins, or oxidation of disulfide bonds to alter protein structure (Alkhalfioui et al., 2007a,b; Oracz et al., 2007). Regardless of the mechanisms causing changes in the dry seed transcriptome with after-ripening, it is important to consider whether changes can impact germination capacity.

The plant hormones abscisic acid (ABA) and gibberellin (GA) act antagonistically to regulate seed dormancy and germination (reviewed in Finkelstein et al., 2008). While ABA promotes seed dormancy, GA stimulates germination. ABA establishes dormancy during seed maturation (Karssen et al., 1983; Lefebvre et al., 2006; Okamoto et al., 2006), while GA biosynthesis and signaling are required for Arabidopsis seed dormancy loss and germination (Koornneef and van der Veen, 1980; Steber et al., 1998; Iuchi et al., 2007; Willige et al., 2007; Hauvermale et al., 2015). ABA-insensitive or biosynthesis mutants rescue the failure to germinate in GA biosynthesis or GA-insensitive mutants (Karssen and Laçka, 1986; Steber et al., 1998). Thus, GA acts upstream of ABA to stimulate germination.

Gibberellin stimulates seed germination, stem elongation, and flowering by negatively regulating the DELLA (Asp-Glu-Leu-Leu-Ala) repressors of GA responses (reviewed in Hauvermale et al., 2012). GA-binding stimulates the protein–protein interaction between the GID1 (GA-INSENSITIVE DWARF1) GA receptors and DELLA protein. Formation of the GID1-GA-DELLA complex causes either DELLA inactivation or destruction via the ubiquitin-proteasome pathway (McGinnis et al., 2003; Dill et al., 2004; Ariizumi et al., 2008, 2011, 2013; Wang et al., 2009; Ariizumi and Steber, 2011). The Arabidopsis *SLEEPY1* (*SLY1*) gene encodes the F-box subunit of an SCF (Skp, Cullin, F-box) E3 ubiquitin ligase that directly binds to and ubiquitinates DELLA upon formation of the GID1-GA-DELLA complex. Thus, GA causes SCF^SLY 1^ to polyubiquitinate, and thereby, target DELLA for destruction by the 26S proteasome. Arabidopsis has five DELLA proteins, *RGA* (*REPRESSOR OF GA1-3*), *GAI* (*GA-INSENSITIVE1*), *RGL1*, *RGL2*, and *RGL3* (*RGA-LIKE*). The failed seed germination of the GA biosynthesis mutant *ga1-3* in the light was strongly rescued by loss of the DELLA *RGL2* (Cao et al., 2005). However, rescue of *ga1-3* germination in the dark, also required loss of DELLAs *RGA* and *GAI*. The GA-insensitive gain-of-function mutation *gai-1* was associated with reduced GA sensitivity during germination in the dark, and reduced germination on ABA in the ABA-insensitive *ABI1-1* mutant background (Koornneef et al., 1985; Ariizumi et al., 2013). DELLAs are thought to repress GA responses through transcriptional regulation via interaction with DNA-binding proteins such as PHYTOCHROME-INTERACTING FACTORS, PIF3, PIF4, and PIF1.

Loss of *SLY1* leads to overaccumulation of DELLA repressors of seed germination associated with increased seed dormancy (Steber et al., 1998; McGinnis et al., 2003; Ariizumi and Steber, 2007). The Arabidopsis GA-insensitive *sly1-2* mutation is a 2-bp deletion leading to loss of the last 40 amino acids of the 151 amino acid protein. Seeds of *sly1-2* have strong initial seed dormancy, but acquire the ability to germinate either with *GID1* gene overexpression (*GID1-OE*) or with 1–2 years of dry after-ripening (Ariizumi and Steber, 2007; Ariizumi et al., 2013). In contrast, Landsberg *erecta* (L*er*) wild-type seeds fully after-ripen within 2 weeks. Neither after-ripening nor *GID1-OE* result in reduced accumulation of DELLA repressors of seed germination. Thus, GA signaling can occur without DELLA-proteolysis leading to increased germination potential. There are three GA receptor genes in Arabidopsis, *GID1a*, *GID1b*, and *GID1c*. *GID1b* protein has higher affinity for GA_4_ and for DELLA protein than GID1a and GID1c (Nakajima et al., 2006; Yamamoto et al., 2010). This is likely the reason that *GID1b-OE* rescues *sly1-2* seed germination and plant height phenotypes better than *GID1a-OE* and *GID1c-OE* (Ariizumi et al., 2008, 2013; Hauvermale et al., 2014).

This paper examines the pattern of transcript accumulation in dry seeds associated with increased seed dormancy and dormancy loss in the GA-insensitive *sly1-2 (sleepy1-2)* mutant of Arabidopsis. Transcripts involved in protein translation were *sly1*-up-regulated in dry seeds, and *sly1*-down-regulated upon seed imbibition. Thus, it appears that *SLY1* may be needed both to down-regulate protein translation during seed development, and to up-regulate translation during germination. The importance of protein translation during seed germination has been well characterized (Galland et al., 2014; Layat et al., 2014). This agrees with our previous research showing that increasing germination capacity with after-ripening is associated with increased abundance of protein translation-associated genes (Nelson and Steber, 2017). In that study, the transcriptional changes associated with *sly1-2* dormancy and dormancy loss were quite different during early and late Phase II of seed imbibition. Based on this result, we postulated that earlier transcriptome differences most likely regulate whether a seed can or cannot germinate. By this rationale, transcriptome differences in dry seeds should play key roles in dormancy and dormancy loss since the stored transcripts in dry seeds are likely the first transcripts to impact germination potential. Consistent with this notion, mutations in two genes showing down-regulation with dry seed after-ripening, the DELLA *GAI* and the histone deacetylase *HDA6*, led to decreased seed dormancy. This suggests that *GAI* and histone deacetylation may establish and maintain seed dormancy.

## Materials and Methods

### Plant Materials and Growth Conditions

*Arabidopsis thaliana* ecotype Landsberg *erecta* (L*er*) wild-type and mutant lines used in this study including *ga1-3*, *sly1-2*, *sly1-2 GID1b-OE*, *gai-1*, *gai-t6*, *sly1-2 gai-t6*, and *sil1* all in the L*er* background were described previously (Peng and Harberd, 1993; Peng et al., 1997; Furner et al., 1998; Steber et al., 1998; Ariizumi et al., 2008). All lines were grown under fluorescent lights in a Conviron^®^ growth chamber according to McGinnis et al. (2003). Harvested seeds were stored at room temperature and low humidity (≈15–30%) in open tubes for dry after-ripening treatments.

The standard practice of harvesting seeds after the entire plant has turned brown (fully desiccated) was used in all cases, except where indicated that harvest occurred at “near maturity.” Since all parts of a plant do not turn brown simultaneously, harvesting fully brown plants means that some portion of the seeds collected have been after-ripening on the plant for up to a few weeks. In order to obtain dormant seeds for wild-type or when expecting germination rates higher than wild-type, seeds were harvested when the mother plants were partially brown and partially green. By collecting only seeds that fell freely from dry siliques and sifting seeds through a fine mesh, we ensured that only brown (desiccated) seeds were collected for use in assays.

#### Microarray Seeds

This study used the same seed batches examined previously during imbibition to investigate starting state transcriptomes of L*er* wt, *sly1-2*(D), *sly1-2*(AR), and *sly1-2 GID1b-OE* (Nelson and Steber, 2017). Two-week-old L*er* wt, *sly1-2*, *sly1-2 GID1b-OE* were grown side-by side, while 19-month-old *sly1-2* was grown in advance to allow comparison of dormant to non-dormant *sly1-2*. All seeds for microarray analysis were collected from fully brown plants. The *GID1b*-overexpression allele in the *sly1-2* background is a translational fusion of *HA:GID1b* on the 35S cauliflower mosaic virus promoter. Growth and storage conditions are described further in Nelson and Steber (2017).

#### L*er* After-ripening Time Course

A single batch of L*er* wt seeds was harvested “near maturity” to collect dormant seeds for an after-ripening time course. Freshly harvested seeds were stored in open tubes overnight before collecting dormant, 0 week after-ripened (0wkAR), seeds for germination and RT-qPCR assays. Seeds from the same batch were collected for RT-qPCR and germination assays each day for 14 days.

#### *GAI* Mutant Germination Assays

Seeds of L*er* wt, *gai-1*, *gai-t6*, and *sly1-2 gai-t6* were grown side-by-side and harvested at near maturity. Freshly harvested seeds were stored in open tubes overnight before collecting dormant 0wkAR seeds for germination assays.

#### *sil1/hda6* Mutant Germination Assay

The *hda6* loss of function mutant in the L*er* background, *sil1* was a kind gift from Dr. Jong-Myong Kim at the RIKEN Plant Science Center in Yokohama, JAPAN. L*er* wt and *sil1* seeds used for germination assays were grown side-by-side and harvested at near maturity to obtain dormant seeds. Freshly harvested seeds were stored in open tubes overnight before collecting dormant 0wkAR seeds for the germination assay. Seeds were stored for an additional 14 days in open tubes then collected for the 2wkAR germination assay.

#### Germination on Tricostatin A

Seeds of L*er* wt, *ga1-3*, and *sly1-2* seeds were harvested from fully brown plants. Seeds were stored for 2 weeks with the exception of long after-ripened *sly1-2*, which was stored for more than 1 year.

### Germination Experiments

For all germination screens, seeds were sterilized with 70% ethanol and 0.01% SDS for 5 min followed by 10% bleach and 0.01% SDS for 10 min, washed, and plated on 0.8% agar plates containing 0.5× MS salts (Sigma–Aldrich) and 5 mM MES [2-(*N*-morpholino)ethanesulfonic acid], pH 5.5 (referred to as MS-agar plates). Germination was scored daily. Germination of the same batch of seeds used for microarray analysis was performed as in Nelson and Steber (2017). For the L*er* after-ripening time course germination of at 0wkAR, 1wkAR, and 2wkAR was scored for three replicates of 100 seeds each after cold stratification for 4 days at 4°C in the dark. L*er* after-ripening time course seeds were the seeds used for the RT-qPCR time course for *AHb1* gene expression in L*er* wt. For the comparison of *GAI* mutants, germination was scored for three replicates of 70–100 seeds each both with and without cold stratification for 4 days at 4°C in the dark. For *sil1* mutants, because we expected higher germination efficiency than wild-type would be difficult to capture, each plate was divided into two halves with L*er* wt plated on one side and *sil1* plated on the other for side-by-side comparison. For the same reason, three replicates of 70 seeds each for each of three biologically independent batches of L*er* wt and *sil1* at 0 and 2 weeks of after-ripening were scored both with and without cold stratification for 4 days at 4°C in the dark. The tricostatin A (TSA) dose response experiments were performed for 2–4 replicates of about 30–90 seeds each. Tricostatin A (TSA) was added to plates at 0, 0.5, 1, 2, 4, and 6 μM concentrations and germination was recorded for 2–4 replicates of about 30–90 seeds.

### Total RNA Isolation from Dry Seeds

RNA extractions for microarray and RT-qPCR were performed as in Nelson and Steber (2017). Briefly, 20 mg of dry seed per sample were flash frozen in liquid nitrogen and RNA was isolated using a phenol-chloroform based extraction method optimized for extraction from tough tissues, such as dry seeds (Nelson and Steber, Unpublished). The extraction method is based on the Oñate-Sánchez and Vicente-Carbajosa (2008) with additional steps to prevent phenol contamination and increase yield. RNA quantity and quality were determined using a NanoDrop ND-2000c spectrophotometer (Thermo Scientific) and gel electrophoresis using RNA denatured at 70°C for 5 min in a formaldehyde dye. For six samples selected from RNA used in the L*er* after-ripening time course RT-qPCR experiment, quality and quality were also determined using the Agilent 2100 bioanalyzer with the RNA 6000 Nano Kit [RNA integrity number (RIN) = 9.0–9.3].

### Microarray and Data Analysis

Microarray analysis of RNA from dry seeds was performed in triplicate using the Affymetrix ATH1 oligonucleotide-based DNA microarray chip (22,810 genes represented). For each replicate of L*er* wt (stored dry for 2 weeks), dormant *sly1-2* (stored dry for 2 weeks), after-ripened *sly1-2* (stored dry for 19 months), and *sly1-2 GID1b-OE* (stored dry for 2 weeks), 2 μg of RNA was processed by the Molecular Biology and Genomics Core Laboratory at Washington State University biotin-labeled cRNA synthesis, ATH1 chip hybridization, and chip scanning^1^. The LIMMA package as part of the Bioconductor suite of tools in the R was used for data analysis as described previously (Gentleman et al., 2004; Smyth, 2005; R Core Team, 2016; Nelson and Steber, 2017). Raw data files are available at ArrayExpress^2^ (Kolesnikov et al., 2015) under accession number E-MTAB-6135. Background correction and normalization was performed by Robust Multi-array Average (RMA), control probesets removed, and significance determined by False Discovery Rate (FDR) with α = 0.05 (Benjamini and Hochberg, 1995; Irizarry et al., 2003).

Reanalysis of published microarray datasets was conducted using the same methods as above to facilitate fair comparison. The raw dataset from Finch-Savage et al. (2007) was obtained from NASCarrays^3^, and dataset from Kendall et al. (2011) was obtained from ArrayExpress. In Finch-Savage et al. (2007) dry seeds of freshly harvested and 120 days after-ripened Cvi wild-type from independent seed batches were analyzed. The Kendall et al. (2011) study compared dry seeds of L*er* wt and *ft-1* collected from dehisced siliques. When referring to the differential regulation in A relative to B, or AvsB, up in AvsB means up-regulated in A (or down-regulated in B), whereas down in AvsB means down-regulated in A (or up-regulated in B).

### Gene Ontology, Gene Family, and TAGGIT Ontology Analyses

Analysis for enrichment in gene categories was performed by (1) looking for global enrichment of genes in standard gene ontology (GO) categories, (2) looking for global enrichment of genes in specific gene families (GF), and (3) looking for enrichment of genes within a specific set of seed dormancy and germination related gene categories (TAGGIT). GO biological process and GF enrichment was performed using the BioMaps tool as part of the VirtualPlant 1.3 suite of online tools for analysis of genomic data^4^ (Katari et al., 2010). Enrichment was determined for a list of differentially regulated genes against the whole genome using a Fisher Exact Test with FDR correction for multiple comparisons using a *p*-value cutoff of *p* < 0.01 (Fisher, 1922). For each significantly enriched category a value for enrichment expected by chance (Expected), was presented for comparison to observed enrichment values (Observed).

For seed germination and dormancy specific GO classifications, the *TAGGITontology* and *TAGGITplot* R functions that we developed previously based on the Carrera et al. (2007) TAGGIT categorizations were used (Nelson and Steber, 2017). These functions are publicly available through github as part of the microarray Tools R package^5^. TAGGIT uses 26 categories defined for their involvement in seed dormancy and germination and matches genes to categories based on lists of AGI locus identifiers in combination with a gene description search for specific keywords. For simplicity, “more up-regulation” or “more down-regulation” in a category refers to a higher degree of enrichment in either the up-regulated gene fraction, or in the down-regulated gene fraction, respectively.

One of the concerns about comparisons of dry seed gene datasets is that differential regulation may be random background due to differences in seed batches. To confirm that the differences in category enrichment identified by TAGGIT could not emerge from a random dataset due to unexpected bias in the computational algorithm, a non-overlapping random set of 330 up- and 430 down-regulated genes was analyzed by TAGGIT (Supplementary Figure 1). This random dataset showed low category enrichments and only small changes between up- and down-regulation datasets, indicating that the differential enrichment in TAGGIT categories observed for *sly1-2* and Cvi dry seed datasets were non-random.

### Transcription Factor Gene Identification in R

To determine the number of transcription-factor-coding mRNAs (TF-mRNAs) in a given geneset a list of Arabidopsis transcription factors was compiled based on the combined databases of PlnTFDB^6^, AtTFDB^7^, and PlantTFDB^8^, since each database contained some unique entries (Supplementary Table 1; Davuluri et al., 2003; Palaniswamy et al., 2006; Pérez-Rodríguez et al., 2009; Zhang et al., 2014). This list contains both true DNA-binding transcription factors and transcription co-factors. In order to categorize a list of TF-mRNAs into transcription factor families, an R function called *countTFs* was written for this study (Supplementary Figure 2). *countTFs* is available for public use as part of the microarrayTools R package through github^9^.

### PlantGSEA Transcription Factor Target Analysis

The web-based Plant GeneSet Enrichment Analysis toolkit (PlantGSEA^10^) with the Transcription Factor Targets (TFT) dataset was used to determine enrichment for known targets of transcription factors within differentially regulated genesets (Yilmaz et al., 2010; Lai et al., 2012; Yi et al., 2013). This toolkit uses published ChIP-seq or ChIP-chip data to identify “Confirmed” or “Unconfirmed” transcription factor targets. Targets that are “unconfirmed” were only identified by a single experimental approach, while “confirmed” targets were identified by two or more approaches with *in vivo* evidence. The “All” category includes both confirmed and unconfirmed targets. Enrichment of transcription factor targets was determined using a Fisher statistical test with the Yekutieli (FDR under dependency) correction for multiple testing adjustment with α = 0.05 (Fisher, 1922; Benjamini and Yekutieli, 2001). To prevent falsely high enrichment for transcription factors with few known targets a 5 hit minimum cutoff was used.

### RT-qPCR Analysis

RT-qPCR analysis was performed using gene-specific primers for *GAI*, *HDA6*, *DOG1*, *SLY1*, *MFT*, *HSFA9*, and *AHb1* for comparison to microarray results. RT-qPCR was also performed for L*er* wt dry seeds at 0, 2, and 4 weeks of after-ripening to determine if an increase in *AHb1* mRNAs could be seen with after-ripening. Primers for *SLY1* were selected to allow binding of both the *sly1-2* mutant and native *SLY1* transcript, since the ATH1 chip cannot distinguish between *SLY1* and *sly1-2* transcripts. The ProScript^®^ M-MuLV First Strand cDNA synthesis kit (New England Biolabs) was used for cDNA synthesis from 1 μg of total RNA and the LightCycler FastStart DNA Master SYBR Green I kit (Roche) was used for qPCR. The QuantPrime online tool^11^ was used for primer design with the exception of the previously published *DOG1*, *GAI*, and *HSFA9* (Zhang and Zhu, 2011; Nomoto et al., 2012; Guan et al., 2013). Primer sequence and annealing temperatures are presented in Supplementary Figure 3. Dilution curves were used to calculate reaction efficiencies; all efficiencies were within 10% of each other and ±10% of 100% efficiency. qPCR conditions were: 10 min at 95°C (initial denature), then 45 cycles of 10 s at 95°C (denaturation), 5 s at the primer-specific annealing temperature (see Supplementary Figure 3), and 10 s at 72°C (extension). Data was analyzed using the Delta–Delta *C*t method with three replicates per gene or timepoint using the AKR2B (*ANKYRIN REPEAT-CONTAINING 2B*; *At2g17390*) reference gene (Livak and Schmittgen, 2001; Hruz et al., 2011). Statistical testing was performed by pairwise *t*-test with Bonferroni–Holm correction for multiple comparisons with α = 0.07 (Supplementary Figure 4; Holm, 1979).

## Results

### Strategies for Examining Mechanisms of *sly1-2* Dormancy and Dormancy Loss in Dry Seeds

**FIGURE 1:**
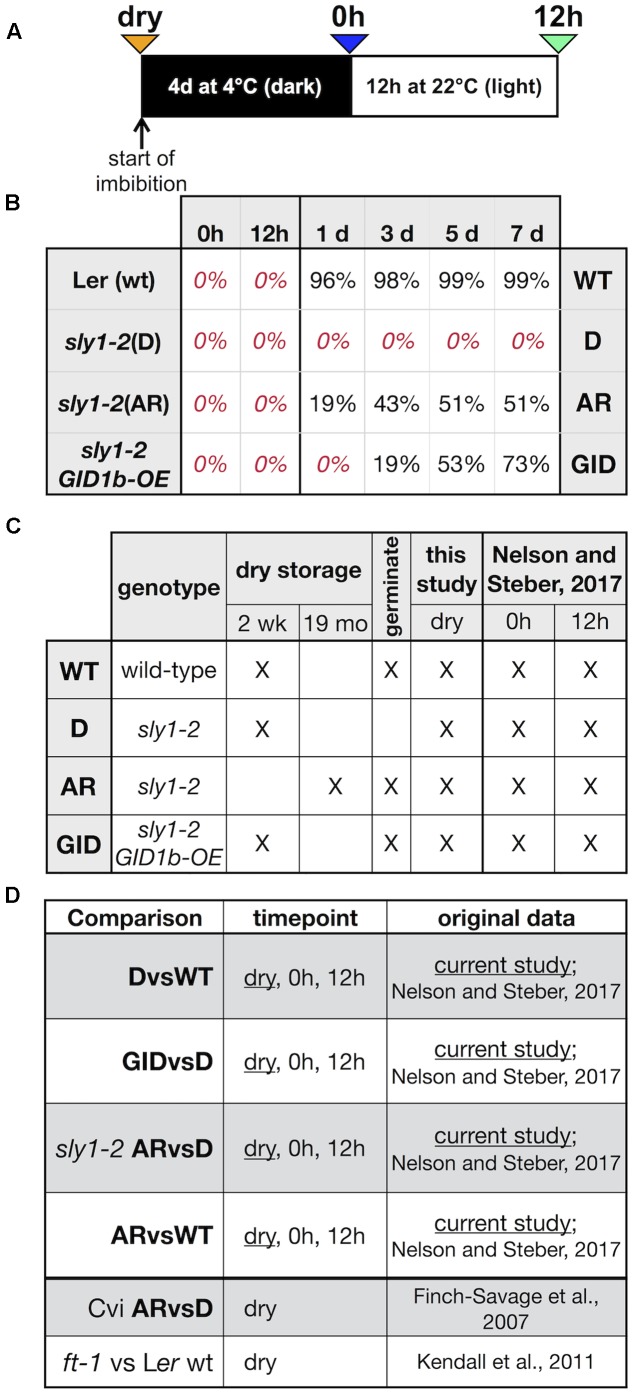
Microarray experimental design. **(A)** Seeds in this study were examined at the “dry” (orange) timepoint in dry seeds. Comparisons were also made to Nelson and Steber (2017) “0h” (blue) timepoint after cold stratification in the dark for 4 days at 4°C and the “12h” (green) timepoint with cold stratification and 12h in the light at 22°C. **(B)** Germination of seeds used for microarray analysis. The same batches of seeds as in Nelson and Steber (2017) were imbibed on MS-agar plates for 4 days at 4°C, then moved to the light at 22°C and scored for germination. **(C)** L*er* wt, *sly1-2*(D), and *sly1-2 GID1b-OE* seed batches were 2 weeks old, while *sly1-2*(AR) was 19 months old seed. **(D)** Experimental comparisons made in this paper, including comparisons from reanalysis of data from Finch-Savage et al. (2007) and Kendall et al. (2011).

In order to ask specific questions regarding the initial transcriptome state of dormant and non-dormant *sly1-2* seeds, an Affymetrix^®^ oligonucleotide-based microarray transcriptome analysis was conducted on dry seeds of: (a) wild-type L*er* (WT) stored for 2 weeks, (b) dormant *sly1-2* stored for 2 weeks [*sly1-2*(D)], (c) after-ripened *sly1-2* stored for 19 months [*sly1-2*(AR)], and d) *sly1-2 GID1b-overexpressed* (*sly1-2 GID1b-OE*) stored for 2 weeks (**Figure 1C**). L*er* WT reached 96% germination after 1 day, whereas *sly1-2*(D) did not germinate even after 7 days of imbibition (**Figure 1B**). *sly1-2* germination was rescued by long after-ripening for 19 months (51% germination by 7 days), and by *GID1b-OE* (73% by 7 days). The same seed stocks were previously used in an imbibed seed microarray study, including a “0h” timepoint taken immediately after cold stratification for 4 days at 4°C in the dark, and a “12h” timepoint (4 days at 4°C, followed by 12h at 22°C in the light) (**Figure 1A**; Nelson and Steber, 2017). Time points examined and comparisons made between this and previous studies are summarized in **Figures 1C,D**.

### Stored mRNA Transcriptome Differences Associated with the *sly1-2* Dormancy Phenotype

**FIGURE 2:**
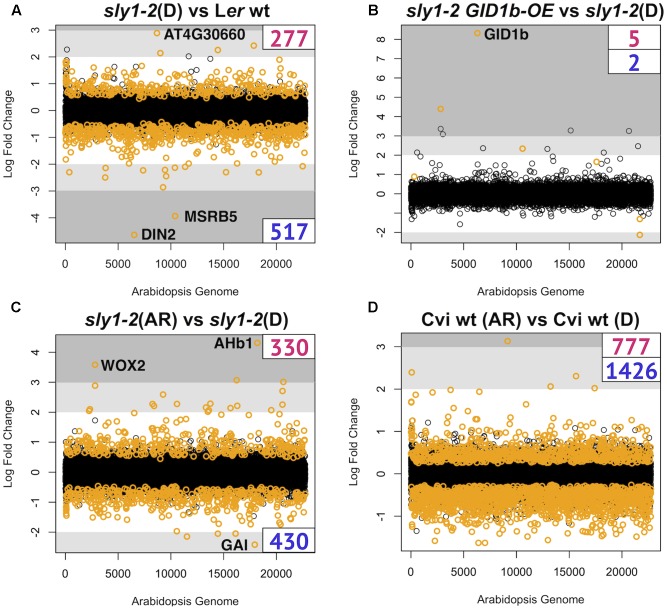
Genome wide expression plots. Plots indicate skew, chromosomal distribution, and magnitude of **(A)** dry seed *sly1*-regulated transcriptome differences, **(B)** dry seed *sly1-2 GID1b-OE* vs. *sly1-2*(D) differences, **(C)** differences after-ripened (AR) and dormant (D) *sly1-2* dry seeds *sly1-2*, and **(D)** differences between after-ripened (AR) and dormant (D) Cvi. Genes with significant differences are indicated in orange (based on FDR *p* < 0.05). Up-regulation is indicated by positive log_2_-fold change (logFC) and down-regulation with negative. Shaded area mark the ±2 and ±3 logFC to allow comparison of magnitude and skew between genesets.

**FIGURE 3:**
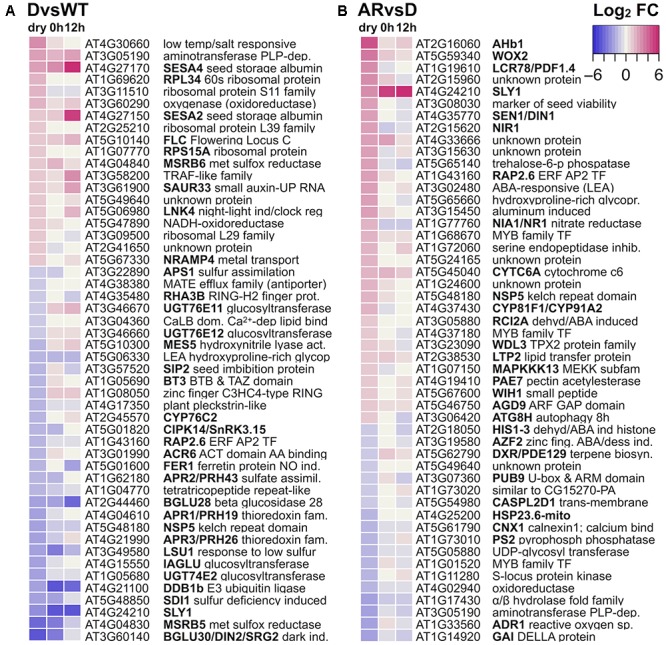
The top 50 largest log_2_-fold change differences in and their transcriptome differences in imbibed seeds. Differences are plotted as a heat map of dry seed values with comparison to the same comparison at 0h and 12h imbibition timepoints from Nelson and Steber (2017). **(A)** Between *sly1-2*(D) and L*er* wt dry seeds (DvsWT) and **(B)** between after-ripened and dormant *sly1-2* (ARvsD) dry seeds. Throughout this work, up-regulation is indicated in red and down-regulation in blue.

The *sly1-2*(D) to wild-type L*er* (*sly1-2* DvsWT) comparison identified 794 transcript differences associated with the *sly1-2* seed dormancy phenotype (**Figure 2A**). Since the comparison of another mutation affecting germination, *ft-1* (*flowering locus t-1*), to L*er* wt dry seeds detected no transcriptome differences (Chiang et al., 2009; Kendall et al., 2011), these changes in dry seed transcript levels were likely effects of the *sly1* mutation during seed development, maturation, or during the 2 weeks of dry after-ripening. The *sly1-2* DvsWT comparison had more negative log_2_-fold changes (logFCs) (517 *sly1*-down-regulated) than positive (277 *sly1*-up-regulated) (**Figure 2A**), resulting in an adjusted Fisher-Pearson standardized moment coefficient skewed toward down-regulation (G1 = -0.56, vs. G1 = 0 if symmetrical) (Joanes and Gill, 1998). Plots comparing normalized intensities showed transcriptome differences across a wide range of signal intensities, indicating that significance was not an artifact of small changes at low intensities (Supplementary Figure 5A). The *sly1-2* F-box mutation results in an inability to degrade DELLA transcriptional regulators (Nelson and Steber, 2016). Thus, negative DELLA regulation in *sly1* mutants may directly or indirectly cause the reduced accumulation of many transcripts during dry seed development. Not surprisingly, some of the top 50 differentially regulated genes were seed-related genes such as a LEA and seed storage proteins (**Figure 3A**). Of the top 50 DELLA/*sly1*-regulated genes in dry seeds, 21 were similarly regulated at the previously published 0h and 12h imbibed timepoints (Nelson and Steber, 2017).

The differentially abundant genes in the dry seed *sly1-2* DvsWT comparison were characterized using BioMaps GO and gene family (GF) to look for biological process enrichment^12^ (Supplementary Figures 6A–C; Katari et al., 2010). There was significant up-regulation of two ribosomal GF, and down-regulation of the glycosyltransferase gene family, including genes involved in auxin and ABA hormone signaling (Supplementary Figure 6A; Yonekura-Sakakibara, 2009). Many *sly1*-up-regulated GO categories were also related to protein translation, ribonucleoprotein complex and ribosome biogenesis (Supplementary Figure 6B). The *sly1*-down-regulated GO categories included stress or stimuli responses related to seed dormancy such as response to ABA, abiotic stress, and oxidation/reactive oxygen species (Supplementary Figure 6C; reviewed in Graeber et al., 2012).

### Transcriptome Differences Associated with Rescue of *sly1-2* Germination by Long After-ripening and *GID1b-OE*

The fact that *sly1* mutants have increased seed dormancy suggests that *SLY1*-directed DELLA destruction is needed for dormancy loss and germination. However, the germination of *sly1-2* seeds is partly rescued by *GID1* overexpression and by long after-ripening without any decrease in DELLA protein accumulation (**Figure 1B**; Ariizumi and Steber, 2007; Ariizumi et al., 2008). We previously learned that *sly1-2* rescue by *GID1b-OE* was associated with far fewer changes in expression than rescue by long after-ripening in imbibing seeds (Nelson and Steber, 2017). We made a similar observation in dry seeds (**Figures 1B,C** and **Table 1**). There were 770 genes with different transcript abundances between D and AR *sly1-2* dry seed, 330 up-regulated and 430 down-regulated with after-ripening of *sly1-2* (*sly1-2* ARvsD). In contrast, only 7 genes showed differential accumulation with *GID1b*-overexpression in *sly1-2* (GIDvsD) dry seeds (**Figure 2B**).

**Table 1:** Complete table of *sly1-2 GID1b-OE* vs. *sly1-2*(D) differentially regulated genes across all three imbibition timepoints.

ID	Gene	dry^a^	0h^a^	12h^a^
At3g63010	GID1b	8.32	8.58	8.28
At5g59310	LTP4	4.40	–	–
At4g02380	LEA5	2.34	–	–
At1g21630	EF hand family	1.65	2.77	2.95
At1g44575	NPQ4	0.89	–	–
At5g46050	PTR3	–	1.26	–
At5g54070	HSFA9	–	–	1.34
At4g09610	GASA2	–	–	1.29
At3g45970	EXPL1	–	–	1.01
At2g34740	A PP2C	–	–	0.97
At3g22490	A LEA	–	–	0.91
At5g45690	Unknown protein	–	–	0.83
At2g46240	BAG6	-2.13	-2.69	-3.19
At2g46250	Myosin heavy chain related	-1.31	-1.80	-3.39
At1g17430	α/β hydrolase fold family	–	-1.10	–
At5g01740	NTF2 family	–	-1.07	–
At5g48850	SDI1	–	-1.06	–
At5g58860	HORST	–	-1.00	–
At1g09200	Histone H3.1	–	-0.93	–
At1g22760	PAB3	–	-0.90	–
At5g56580	ANQ1/MKK6	–	-0.83	–
At1g56190	Phosphoglycerate kinase	–	-0.79	–
At5g15230	GASA4^b^	–	–	-1.51
At5g07480	KUOX1	–	–	-1.28
At2g44800	Oxidoreductase	–	–	-1.13
At2g40880	CYSA	–	–	-0.77
At2g16060	AHb1/GLB1	–	–	-0.73

^a^*sly1-2 GID1b-OE sly1-2 (D) log_*2*_ fold changes*.

^b^*log_2_ fold change of -1.51 ± 0.56, significant based on RT-qPCR*.

While more transcripts showed decreased rather than increased levels with after-ripening, the dataset was slightly skewed toward AR-up-regulation (G1 = 0.35), likely due to stronger up-regulation of fewer transcripts (**Figure 2C**). For example, there were 20 up-regulated transcripts with logFCs from 2 to 4.3, whereas only 4 of the down-regulated transcripts had logFCs greater than 2. This is consistent with observations made during dry after-ripening of the dormant ecotype Cvi; where there were 777 up- and 1426 down-regulated transcripts in the Cvi ARvsD comparison (**Figure 2D**). Since the plotted normalized intensities of *sly1-2* ARvsD showed significant differences (red) over a wide range of intensities, the small number of transcripts highly up-regulated do not appear to be artifacts of comparing low intensity values (Supplementary Figure 5B). Many of the *sly1-2* ARvsD transcriptome changes observed in dry seeds were also seen at 0h and 12h of imbibition, but with lower logFCs (**Figure 3B**). The most up-regulated gene was the *AHb1* (*Arabidopsis nonsymbiotic Hemoglobin1*; Abbruzzetti et al., 2011) gene involved in oxidative stress response, whereas the most down-regulated gene was the DELLA *GAI*. It is interesting that *GAI* was up-regulated in the *sly1-2* DvsWT dry seed comparison and down-regulated with dry after-ripening (**Figure 3B**). This suggests that *GAI* plays a role in *sly1-2* dormancy that is reversed with long after-ripening. BioMaps gene family analysis and GO analysis showed that many of the dry seed *sly1*-regulated terms (*sly1-2* DvsWT) were oppositely AR-regulated (Supplementary Figures 6A–C; Katari et al., 2010). The *sly1*-down-regulated stimuli response terms, including ABA and abiotic stress, were AR-up-regulated in dry *sly1-2* seeds (Supplementary Figure 7A). Only translation and terms related to cellular/metabolic processes were *sly1*-up- and AR-down-regulated (Supplementary Figures 6A,B, 7B).

**FIGURE 4:**
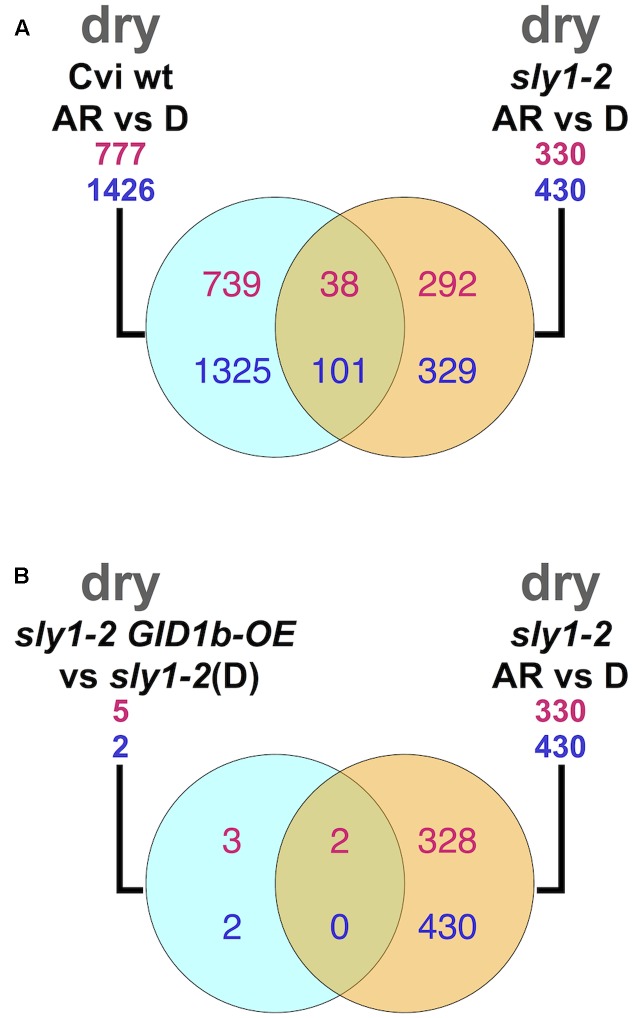
Comparisons between dry transcriptome datasets. **(A)** The overlap between *sly1-2* and Cvi dry seed after-ripening regulated datasets. Cvi dataset is from Finch-Savage et al. (2007). **(B)** The overlap between dry seed *GID1b-OE*-regulated and after-ripening regulated mRNAs.

The significant overlap between AR-regulated genes in Cvi and *sly1-2*, despite the fact that *sly1-2* is in the L*er* ecotype, suggests that these changes are biologically relevant (**Figure 4A**). The direct overlap of *sly1-2* and Cvi AR-regulated transcriptome changes identified a list of genes associated with both Cvi wt and *sly1-2* dormancy loss (Supplementary Table 2). There were 38 up- and 101 down-regulated transcripts in *sly1-2* and Cvi with after-ripening. This smaller dataset included genes that are AR-regulated in both *sly1-2* and Cvi wt. This dataset included many genes related to ABA or GA signaling and germination. Among them, the DELLA *GAI*, 5 members of the ABA PP2C (Protein Phosphatase Type 2C) family genes, *MFT* (*MOTHER OF FT AND TFL*), and *HDA6* (*HISTONE DEACETYLASE6*) were all AR-down-regulated.

*GID1b-OE* rescue of *sly1-2* germination was associated with only seven differentially abundant transcripts in dry seeds, 5 up- and 2 down-regulated (**Table 1**). Since *GID1b* is overexpressed on the 35S promoter, it was not surprising that the most up-regulated gene was *GID1b* itself. Among the remaining 6 genes, 3 were similarly regulated at 0h and 12h of imbibition, including: the up-regulated *At1g21630* (EF hand family) gene, and down-regulated *At2g46250* (myosin heavy-chain related) and *BAG6* (*BCL-2-Associated Anthogene6*) genes. When the dataset was compared to the dry seed transcriptome changes with after-ripening of *sly1-2*, *LTP4* and *LEA5/SAG21* were *GID1b-OE*- and AR-up-regulated (**Figure 4B**). *LTP4* encodes a phospholipid transfer protein localized to the cell wall, while *LEA5/SAG21* encodes a senescence-associated protein with a role in oxidative stress tolerance (Arondel et al., 2000; Hundertmark and Hincha, 2008). Both *LTP4* and *LEA5/SAG21* are also ABA-induced transcripts.

### Protein Translation and Gene Transcription Are Major Gene Categories Regulated by *SLY1* and After-ripening

**FIGURE 5:**
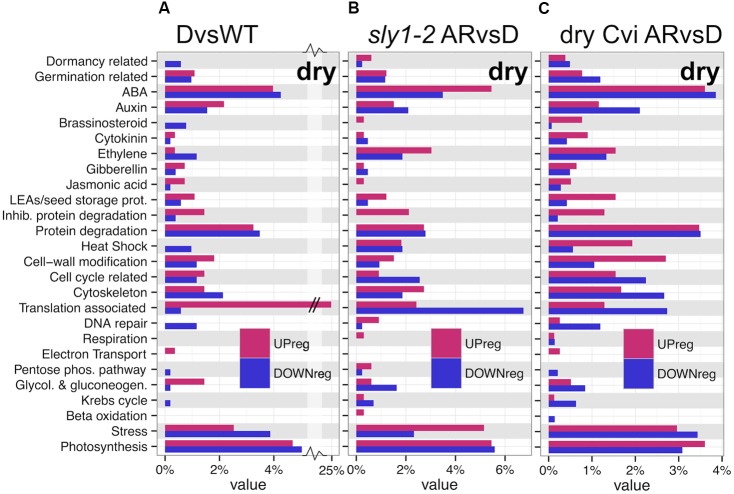
TAGGIT gene ontology analysis of *sly1-* and after-ripening-regulated transcriptome differences in dry seeds. **(A)** *sly1-2* DvsWT dry seed transcriptome differences. **(B)** Differences with after-ripening of *sly1-2* dry seeds. **(C)** Differences with after-ripening of Cvi dry seeds. The value on the *x*-axis shows the percentage of either the total up-regulated or total down-regulated genes within a dataset.

TAGGIT seed-related ontology analysis was used to compare gene enrichment in seed-specific categories for genes differentially regulated in DvsWT, *sly1-2* ARvsD (current study, L*er* ecotype), and ecotype Cvi ARvsD dry seed comparisons (**Figures 1D**, **5**; Carrera et al., 2007; Finch-Savage et al., 2007; Nelson and Steber, 2017). It is interesting that the protein translation category accounted for 25% of the *sly1*-up-regulated genes (DvsWT; **Figure 5A**) given that the translation category was among the most highly *sly1*-down-regulated at 0h and 12h of seed imbibition in our previous study (Supplementary Figure 8; Nelson and Steber, 2017). The translation category was also strongly down-regulated with after-ripening of both *sly1-2* and Cvi dry seeds (**Figures 5B,C**). In contrast, the translation category showed strong up-regulation with after-ripening of imbibed L*er* wt but not *sly1-2* seeds (Nelson and Steber, 2017). Thus, it appears that the *SLY1* gene is needed both to down-regulate protein translation-associated genes during seed development and to up-regulate protein translation genes during seed germination.

It appears that dry after-ripening involves similar mechanisms in *sly1-2* and Cvi since many TAGGIT categories, such as auxin, ethylene, LEAs, inhibition of protein degradation, cell wall, and cell cycle, showed similar regulation in both experiments (**Figures 5B,C**). TAGGIT analysis of a randomly generated dataset confirmed that TAGGIT profiles similar to those observed for *sly1-2* ARvsD and Cvi ARvsD were unlikely to happen by chance, suggesting that this agreement has functional relevance (Supplementary Figure 1). However, there was not perfect agreement in all *sly1-2* and Cvi categories. For example, ABA was strongly up-regulated in *sly1-2*, but slightly down-regulated in Cvi, while the cytoskeleton category was up-regulated in *sly1-2* but down-regulated in Cvi. Since these categories were similarly regulated in *sly1-2* and L*er* during late Phase II, they may result from either the *sly1* mutation or ecotype differences (Nelson and Steber, 2017).

The first proteins translated from stored mRNAs may activate or block transcriptional cascades leading to germination. Thus, we examined if differentially expressed transcription-factor-encoding mRNAs (TF-mRNAs) are among the AR-regulated genes in dry seeds using a combined list of Arabidopsis transcription factors compiled from the PlnTFDB, AtTFDB, and PlantTFDB databases (Davuluri et al., 2003; Palaniswamy et al., 2006; Pérez-Rodríguez et al., 2009; Jin et al., 2013). This analysis revealed 27 transcription-factor-encoding mRNAs (TF-mRNAs) up-regulated and 42 TF-mRNAs down-regulated with dry after-ripening (Supplementary Figure 9C). Categorization of genes by transcription factor families using the *countTFs* R function, written for this study (see Section “Materials and Methods”), revealed that transcription factor families strongly regulated with *sly1-2* after-ripening included AP2-EREBP, ARF (Auxin Response Factors), C3H (Cys3His zinc fingers), GRAS, and MYB-related families (Supplementary Figure 9D).

**FIGURE 6:**
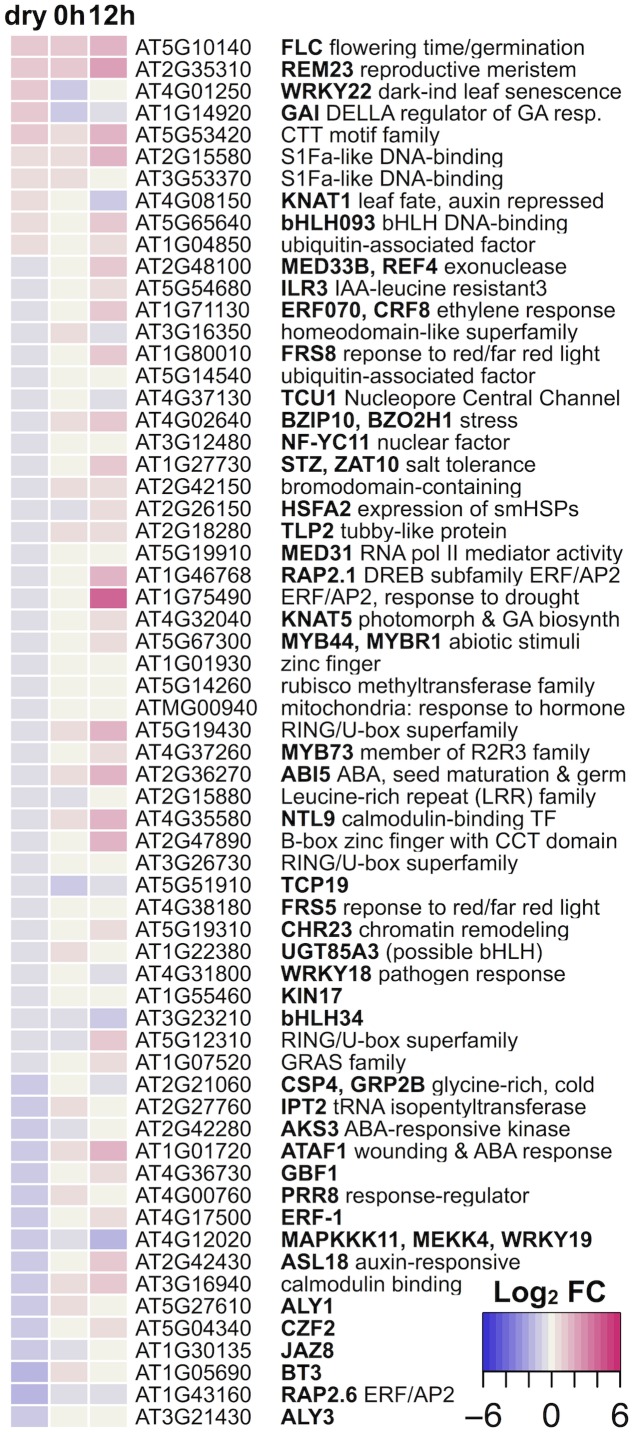
Heat map of all dry seed *sly1-2* DvsWT differentially regulated transcription factors showing their expression changes in dry seeds, at 0h, and at 12h of imbibition.

Since 2 weeks of dry after-ripening is sufficient to stimulate wild-type L*er* but not in *sly1-2* germination, we examined changes in TF-mRNA accumulation in the *sly1-2* DvsWT dry seed comparison. Of the 794 *sly1*-regulated transcripts, 53 TF-mRNAs were *sly1*-down-regulated, while only 10 TF-mRNAs were up-regulated (**Figure 6** and Supplementary Figure 9A). Thus, a major effect of the *sly1* mutation appears to be loss of TF-mRNAs that may be translated during imbibition. When these TF-mRNAs were examined at 0h and 12h, most of the dry seed *sly1*-down-regulated genes were not similarly regulated at 0h or 12h, while 7 of the 10 *sly1*-up-regulated genes were similarly regulated at 0h or 12h of imbibition (**Figure 6**). The *sly1*-down-regulated TF-mRNAs families included AP2-EREBP (APETALA2 and ethylene-responsive element binding proteins), bHLHs (basic helix-loop-helix), C2H2 zinc fingers, and MYB-related family transcription factors (Supplementary Figure 9B). The DELLA *GAI* was among the *sly1*-up-regulated TF-mRNAs. Thus, DELLA accumulation in *sly1-2* may promote *GAI* expression, possibly through feed-forward regulation (Zentella et al., 2007).

In addition to TF-mRNAs, the Plant GeneSet Enrichment Analysis (PlantGSEA) tool was used to look for enrichment of known transcription factor targets within the dataset of stored mRNA differences in the *sly1-2* DvsWT dry seed comparison (Yilmaz et al., 2010; Lai et al., 2012; Yi et al., 2013). Targets of the bHLH transcription factor PIF1/PIL5 (PHYTOCHROME INTERACTING FACTOR1/PIF3-LIKE5) were strongly enriched in the *sly1*-down-regulated geneset, representing 9% of the *sly1*-down-regulated genes in dry seeds (Supplementary Figure 10). Thus, PIF1/PIL5 may represent a *SLY1*-dependent regulator of seed dormancy.

### An Association between mRNA Stability and Changes in Relative Transcript Levels with Dry After-ripening

**FIGURE 7:**
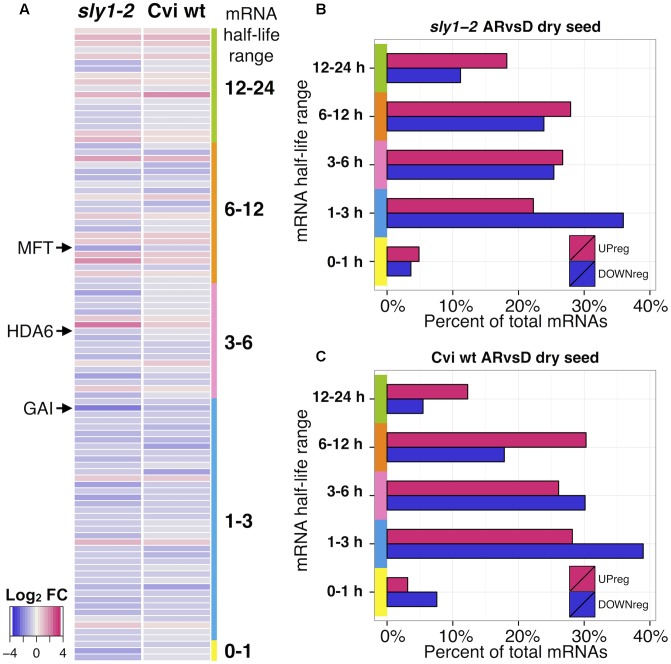
Transcriptome differences categorized based on inherent mRNA stability. **(A)** Heat map of genes differentially regulated in dry seeds with after-ripening of both *sly1-2* and Cvi wt. Genes are plotted in order of high to low mRNA stability determined based on the half-life scores from Narsai et al. (2007). **(B,C)** Plots of fractions of after-ripening-up- and down-regulated genes in each half life range stability category. **(B)** For *sly1-2* dry seed transcriptome changes. **(B)** For Cvi dry seed transcriptome changes. There was a correlation of higher stability with up-regulation and lower stability with down-regulation in dry seed datasets. Both datasets had few genes with half-life in the 0–1 h range.

Seed dormancy is relieved by after-ripening during dry storage. Little metabolic activity is possible in a dry seed, suggesting that differences in transcript turnover rates rather than active transcription may cause the changes in transcript abundances observed with dry after-ripening. Data analysis was used to explore whether apparent up- or down-regulation of stored mRNA was associated with differences in transcript stability. If a small number of stable or protected mRNAs degrade more slowly than the ribosomal RNA, microarray of apparently equal RNA amounts would indicate that these stable genes were up-regulated. A previous study identified genome-wide mRNA stabilities for 13,012 transcripts by measuring transcriptome changes over time after L*er* cell cultures were treated with the transcriptional inhibitor Actinomycin D (Narsai et al., 2007). This included mRNA half-life values for 99 of the 139 *sly1-2* and Cvi AR-regulated transcripts. A heatmap of these 99 AR-regulated transcript changes was plotted in decreasing order of mRNA half-life to examine whether lower intrinsic mRNA stability was associated with decreasing mRNA levels with dry after-ripening (**Figure 7A**). Although mRNA stability alone cannot account for all up- and down-regulation, shorter half-life mRNAs appeared more AR-down-regulated and longer half-life mRNAs appeared more AR-up-regulated. Similarly, when the AR-regulated transcripts were categorized by half-life range, a larger percentage of stable mRNAs (12–24 h or 6–12 h half-life) were up-regulated, whereas more unstable mRNAs (1–3 h half-life) were down-regulated (**Figures 7B,C**). This trend for high stability mRNAs to be up-regulated and lower stability mRNAs to be down-regulated was not seen at *sly1-2* ARvsD 0h and 12h timepoints, indicating that mRNA stability is not the major determinant of transcript levels in imbibing seeds (Supplementary Figures 11A–C). The dry transcriptome counterexamples where mRNA stability was high, yet transcript levels were low or vice versa may be transcripts subject to more active regulation, such as protection by an RNA-binding proteins or targeted mRNA oxidation.

### Comparison of Differential Regulation of Stored mRNAs by RT-qPCR and Microarray

**FIGURE 8:**
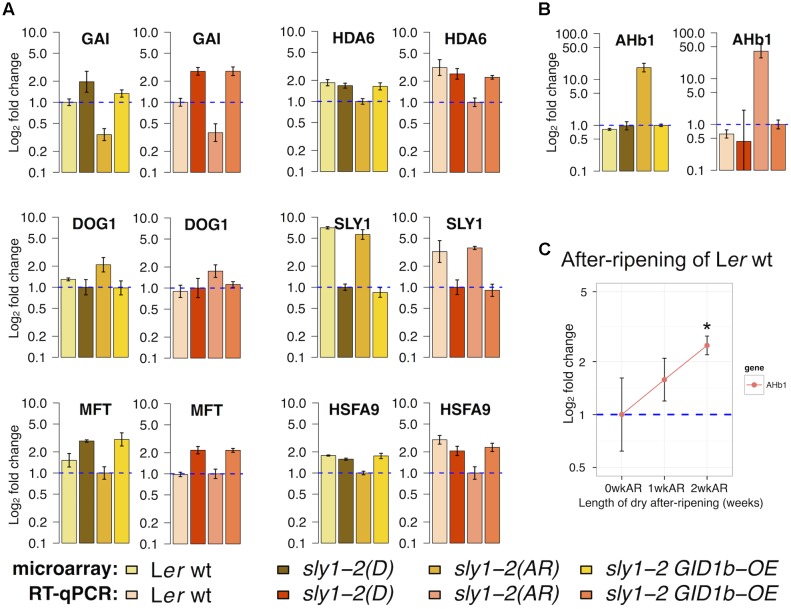
Comparing RT-qPCR analyses of transcriptome differences with those measured by microarray. **(A)** Plots for a selection of genes with differential regulation in both ARvsD and DvsWT comparisons and **(B)** plots for *AHb1*, the most AR-up-regulated gene in *sly1-2* dry seeds. Both microarray (brown) and RT-qPCR (orange) relative expression are shown relative to the same calibrator, set to height of 1 and indicated by the blue dotted-line. For this comparison, RMA normalized microarray data was analyzed using the ddCT method relative to the same constitutively expressed AKR2B control gene used for analysis of RT-qPCR data. **(C)** L*er* wt was harvested “near maturity” and seeds were collected for RT-qPCR to examine transcript levels of *AHb1* at 0, 1, and 2 weeks of after-ripening (0, 1, and 2wkAR). Asterisk indicates significance relative to 0wkAR (*p* = 0.04). For all RT-qPCR experiments, statistical significance was determined by pairwise *t*-test with Bonferroni–Holm correction for multiple comparisons (see Supplementary Figure 4 for *p*-values). Error bars represent SD.

RT-qPCR analysis was used to validate transcript level differences identified by microarray in the *sly1-2* ARvsD and/or DvsWT comparisons (**Figure 8**). For comparison, both RT-qPCR and microarray expression were plotted relative to the constitutively expressed control gene *AKR2B* (*ANKYRIN REPEAT-CONTAINING 2B, At2g17390*) (Hruz et al., 2011). RT-qPCR confirmed that *GAI*, *HDA6*, *MFT*, and *HSFA9* (*HEAT SHOCK FACTOR A9*) were AR-down-regulated, while *GAI* and *MFT* were *sly1*-up-regulated in dry seeds (**Figure 8A**). As in imbibed seeds, the *SLY1*/*sly1-2* transcript was AR-up-regulated and *sly1*-down-regulated in dry seeds (Nelson and Steber, 2017). The dormancy-associated *DOG1* (*DELAY OF GERMINATION1*) gene was AR-up-regulated in the *sly1-2* microarray analysis, but just outside of statistical significance (*p* = 0.071) by RT-qPCR. Conversely, *DOG1* was AR-down-regulated in Cvi wt (Finch-Savage et al., 2007). Finally, the *AHb1* transcript was highly AR-up-regulated based both on microarray and RT-qPCR (*p* = 8 × 10^-4^) analysis in *sly1-2* (**Figure 8B**).

Since *AHb1* was not significantly up-regulated with ecotype Cvi dry after-ripening, it may be the case that AR-up-regulation of *AHb1* is dependent on the L*er* ecotype. Thus, an after-ripening time course examined if *AHb1* was up-regulated with dry after-ripening of wild-type L*er*. RNA was isolated from dry L*er* seeds immediately after harvest at maturity (0 weeks after-ripened, 0wkAR), then after-ripened for 1 (1wkAR) and 2 weeks (2wkAR). *AHb1* mRNA levels showed an increasing trend with AR, and a significant increase from 0wkAR to 2wkAR by RT-qPCR analysis (**Figure 8C** and Supplementary Figure 12). Thus, *AHb1* is up-regulated with dry after-ripening in the L*er* ecotype, both in WT and *sly1-2* seeds.

### Functional Analysis of DELLA *GAI* and *HDA6*, Genes Down-regulated with Dry After-ripening

Dormancy loss due to dry seed after-ripening may result from degradation of transcripts encoding strong negative regulators of seed germination. For example, DELLA family genes are known to negatively regulate Arabidopsis seed germination. Both DELLA *GAI* and the histone deacetylase *HDA6* were down-regulated with dry after-ripening of both *sly1-2* and Cvi seeds. In addition, *GAI* was up-regulated in the *sly1-2* DvsWT dry seed comparison, indicating that *GAI* mRNA expression is associated with seed dormancy and negatively regulated by *SLY1* and after-ripening. To examine whether the down-regulation of these mRNAs with dry after-ripening is functionally relevant, the effect of mutant alleles on seed dormancy and dormancy loss were examined.

**FIGURE 9:**
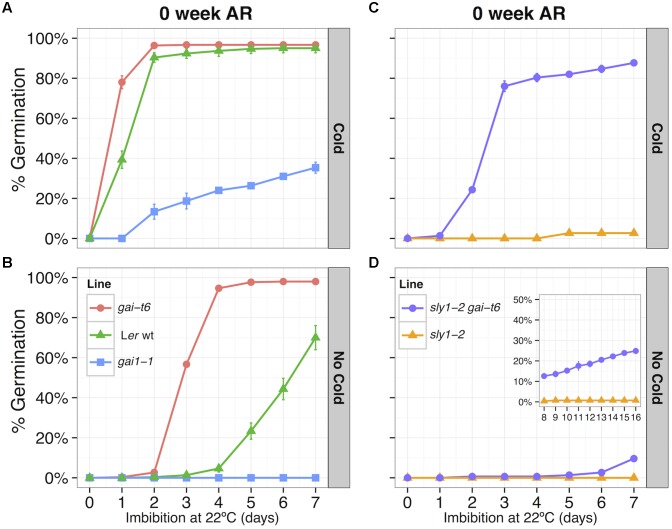
Examining the role of *GAI* in the regulations of seed germination based on germination screens using freshly harvested seeds collected at near maturity. **(A,B)** Comparing of L*er* wt, *gai-1* (gain of function allele), and *gai-t6* germination: **(A)** with cold stratification for 4 days at 4°C, before moving to the light at 22°C where germination was scored daily (“Cold”), and **(B)** without cold, seeds placed directly at 22°C and germination scored daily (“No Cold”). Loss of *GAI* function leads to an increase in germination and gain of *GAI* function leads to increased dormancy. **(C,D)** Comparing *sly1-2* and *sly1-2 gai-t6* germination **(C)** with cold stratification, and **(D)** without cold stratification. Loss of *GAI* function caused partial rescue of *sly1-2* seed germination.

Based on double mutant studies with *ga1-3*, DELLA *GAI* was believed to play a less important role in repressing seed germination than DELLA *RGL2* (Lee et al., 2002; Tyler et al., 2004; Cao et al., 2005). While *RGL2*, *RGL3* and *GAI* transcript levels were high in imbibing WT, *sly1-2(D)*, *sly1-2(AR)*, and *sly1-2 GID1b-OE* seeds, the fact that only *GAI* and *RGL3* transcript levels were high in dry seeds suggests that *GAI* may be more important in dry seed after-ripening (Supplementary Figure 13). Furthermore, *GAI* was the only DELLA transcript differentially regulated with after-ripening in dry *sly1-2* seeds, showing AR-down-regulation in both *sly1-2* and Cvi wt seeds. Consistent with the notion that *GAI* regulates seed dormancy, *gai-t6* had a higher and *gai-1* a lower germination rate than wild-type L*er* seeds when seed germination was examined in highly dormant fresh seeds harvested at near maturity (**Figures 9A,B**). Cold stratification improved germination for all lines, but *gai-t6* consistently germinated faster than wild-type, while *gai-1* germinated slower. If elevated *GAI* mRNA levels in *sly1-2* seeds stimulate dormancy, then we would expect *gai-t6* to rescue *sly1-2* seed germination. Indeed, while dormant *sly1-2* seeds failed to germinate even with cold stratification, the *sly1-2 gai-t6* double mutant germinated without cold stratification reaching 25% with 16 days of incubation (**Figures 9C,D**). Taken together, these results suggest that *GAI* plays an early role in the negative regulation of seed germination.

**FIGURE 10:**
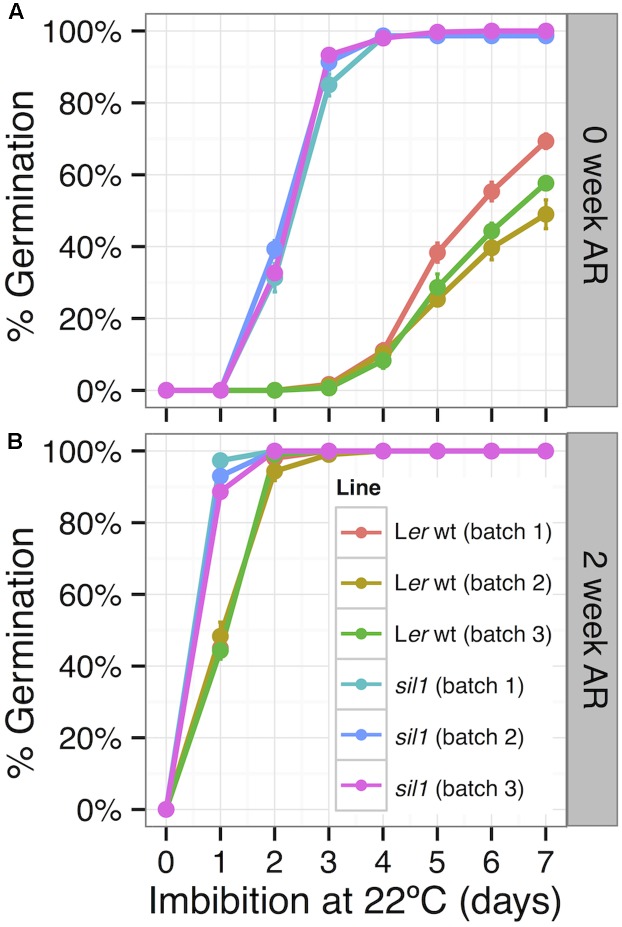
A germination screen was performed to compare germination of *sil1* and L*er* wt harvested at near maturity. Seeds were germinated at two timepoints, **(A)** freshly harvested (0 week AR), and **(B)** 2 weeks old (2 weeks AR). Three biologically independent batches of seed were assayed to clearly capture the *HDA6* loss of function phenotype in *sil1*. To minimize dormancy release, seeds were placed directly at 22°C and germination was scored daily. Freshly harvested *sil1* seed germinated more efficiently than wild-type, both at 0 and at 2 weeks of after-ripening.

If *HDA6* stimulates seed dormancy in wild-type L*er*, then we would expect *hda6* mutants to be less dormant than wild-type. The germination phenotype of the *HDA6* allele in the L*er* background called *sil1* (*modifiers of silencing1*) was examined in seeds harvested near maturity to maximize dormancy. Seeds of *sil1* germinated more efficiently than wild-type L*er* in three biologically independent batches of seeds at 0 and 2 weeks of after-ripening, both with and without cold stratification (**Figures 10A,B** and Supplementary Figures 14A,B). This suggests that histone deacetylation by *HDA6* stimulates seed dormancy, presumably by inhibiting the expression of genes needed for germination.

**FIGURE 11:**
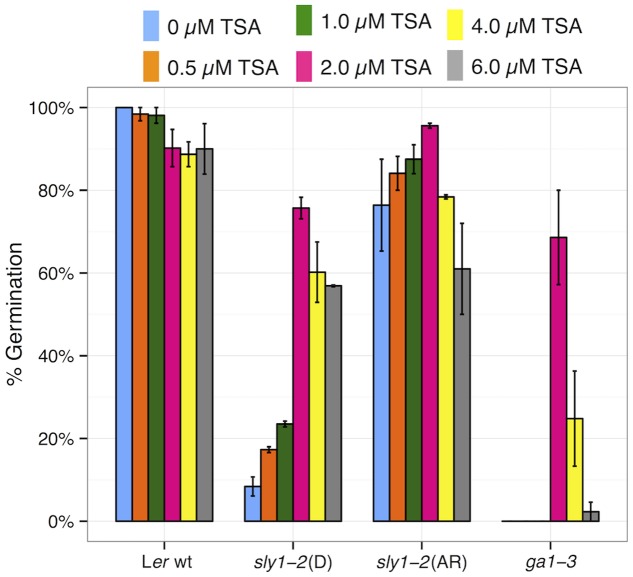
Germination of L*er* wt, *sly1-2*(D), *sly1-2*(AR), and *ga1-3* was conducted on varying concentrations of the histone deacetylase inhibitor, tricostatin A (TSA). TSA stimulated germination of both dormant *sly1-2* and of *ga1-3*. Rescue of germination was most efficient at 2.0 μM TSA.

If histone deacetylation stimulates the seed dormancy of GA mutants, then inhibition of histone deacetylation should rescue the germination of GA-insensitive *sly1-2* and of the GA biosynthesis mutant *ga1-3*. This was examined using a specific inhibitor of histone deacetylases called tricostatin A (TSA) (Yoshida et al., 1995). TSA rescued the germination of dormant and after-ripened *sly1-2* in a dose-dependent manner (**Figure 11**). Interestingly, TSA also stimulated the germination of *ga1-3* seeds, suggesting that GA functions in part by relieving transcriptional repression by histone deacetylases. TSA rescued germination most efficiently at 2 μM (76%), and showed decreasing germination at 4 and 6 μM TSA. It may be that histone deacetylation and TSA alter the expression of other positive or negative regulators of germination at different concentrations.

## Discussion

### DELLA-Directed Seed Dormancy in *sly1-2*

There are many mechanisms contributing to seed dormancy. The *sly1* mutant has increased dormancy due to overaccumulation of DELLA proteins, the negative regulators of GA responses and seed germination. Thus, comparing *sly1-2* vs. WT (DvsWT) defined transcriptome differences associated with DELLA-imposed seed dormancy.

The majority (65%) of these genes were down-regulated in *sly1-2*, suggesting that a major effect of *sly1* loss/increased DELLA is decreased transcript abundance (**Figure 2A**). DELLA proteins act in concert with DNA-binding proteins to regulate transcription (Oh et al., 2004, 2006, 2007; Gallego-Bartolomé et al., 2010). Thus, it is interesting that the DELLA-interactor PIF1/PIL5 is a regulator of many highly *sly1*-down-regulated transcripts (Supplementary Figure 10). PIF-regulated genes were expected to be among *SLY1*/DELLA-regulated genes because DELLA proteins bind PIF3 and PIF4, inhibiting PIF DNA-binding and transcriptional activation while promoting PIF3 protein degradation by the 26S proteasome (de Lucas et al., 2008; Feng et al., 2008; Li et al., 2016). PIF1/PIL5 is a known DELLA interactor whose negative regulation of germination is relieved by light (Oh et al., 2004, 2006, 2007; Gallego-Bartolomé et al., 2010). Thus, it is appears that DELLA overaccumulation in *sly1-2* seeds during development or maturation may cause transcriptional repression of PIF1/PIL5-regulated gene targets accounting for some of the down-regulation of stored mRNAs in dry seeds.

Transcription factors produced early in seed imbibition are ideal candidates to initiate the transcriptional cascades leading to or blocking germination *per se*. There were 5-times more *sly1*-down-regulated than *sly1*-up-regulated TF-mRNAs (Supplementary Figure 9). This suggests that DELLA overaccumulation in *sly1* leads to lower expression of transcription factors. Known regulators of germination, *ABI5* (*ABA-INSENSITIVE5*) and DELLA *GAI* are examples of major *sly1*-regulated TF-mRNAs (**Figure 6**; Koornneef et al., 1985; Lopez-Molina et al., 2002). Thus, different levels of germination-promoting or -inhibiting TFs in *sly1-2* and WT may be one mechanism allowing wild-type L*er*, but not *sly1-2*, seeds to germinate at 2 weeks of after-ripening.

While it is tempting to believe that dry seed transcriptional differences in *sly1-2*(D) compared to WT arise entirely during development or maturation, these differences may also arise during 2 weeks of dry storage. For example, transcripts may be degraded at different rates in different genotypes, either faster or slower in the *sly1-2* mutant than in WT. Since *sly1-2* requires 1–2 years to reach a germination rate similar to WT after-ripened for 2 weeks, it is possible that some germination-inhibiting transcripts require more time to degrade or oxidize in *sly1-2* than in WT. It could also be the case that germination-promoting transcripts are less protected in *sly1-2*. Investigation of DvsWT transcriptome differences during development and maturation might help to differentiate transcriptome differences arising during development from those arising during dry storage.

### Evidence for the Functional Relevance of Dry Seed Transcriptome Changes

While it may be argued that changes in the dry seed transcriptome are merely artifacts of mRNA oxidation/damage over time, the results of this study provide circumstantial evidence that some of these changes are of regulatory importance in dormancy loss. First, similar changes occurred with dry after-ripening in two different ecotypes. Second, transcription factors known to function in dormancy, dormancy loss, and GA signaling were among the AR-differentially regulated genes. And third, mutations in two of these differentially regulated genes resulted in altered seed dormancy and germination.

The overlap in the *sly1-2* and Cvi ARvsD comparisons suggested that dry seed transcriptome changes are not due to random degradation of transcripts as seeds age, but may represent dormancy-loss mechanisms. Of the 770 stored mRNAs that were differentially regulated with after-ripening in dry *sly1-2* seeds, 12% of the AR-up-regulated and 23% of the AR-down-regulated were similarly regulated in Cvi wt (**Figure 4A**). Since *sly1-2* is a mutation in the L*er* rather than the Cvi ecotype, differences between these two ARvsD comparisons may result either from ecotype differences or the *sly1-2* mutation. Interestingly, the regulation of TAGGIT gene categories was similar in *sly1-2* and Cvi wt dry seed after-ripening (**Figures 5B,C**). The partial overlap in the *sly1-2* and Cvi ARvsD comparisons may simply suggest that the seed dormancy of the two genotypes results from only partially overlapping mechanisms. In other words, there are multiple ways to acquire and to lose seed dormancy.

Even transcripts that are AR-regulated in *sly1-2* but not Cvi may function in after-ripening of the L*er* ecotype. For example, the *AHb1* transcript was not AR-up-regulated in Cvi, but was strongly AR-up-regulated transcript in dry seeds of *sly1-2* and L*er*. *AHb1* (also called Arabidopsis class 1 phytoglobin or *pgb1*) protects roots from severe oxidative stress (Hill et al., 2016; Mira et al., 2017). Thus, it may play a similar role in dry seeds. There appears to be a link between class 1 phytoglobin expression and seed dormancy/germination in barley (Ma et al., 2016). Dormancy can also be rescued without a large change at the transcriptome level, as evident by *GID1b-OE* rescue of *sly1-2* seed germination, where only 27 genes were differentially regulated at any of the three timepoints investigated (**Table 1**). Of these, the *AHb1* transcript was down-regulated at 12h of imbibition. Future research will need to examine if *AHb1* is needed to stimulate *sly1-2* germination in early Phase I, but not in Phase II of germination.

Transcription factors produced early in seed imbibition are ideal candidates to initiate the transcriptional cascades leading to or blocking germination *per se*. Thus, it is interesting that transcription factors known to control dormancy and dormancy loss were among the AR-regulated genes. ABA hormone establishes dormancy, ethylene can break dormancy in *ga1-1*, and auxin has been implicated in dormancy and dormancy release (Finkelstein et al., 2008; Karssen et al., 1989). In light of this, it is interesting that TAGGIT ontology analysis found that 9% of TFs were ABA-related, 12% were ethylene-related, and 7% were auxin-related (Supplementary Figure 9C). For example, ABA related protein phosphatase genes, *HAB2* (*HOMOLOGY TO ABI2*), *AHG3* (*ABA-HYPERSENSITIVE GERMINATION3*), and *HAI3* (*HIGHLY ABA-INDUCED PP2C GENE3*) were among transcripts down-regulated with *sly1-2* after-ripening (Supplementary Table 2; Finkelstein et al., 2008). Moreover, the negative regulator of germination and GA signaling, DELLA *GAI* was also AR-down-regulated in dry *sly1* seeds (**Figure 6**; Koornneef et al., 1985). Examination of mutations in two *sly1* AR-downregulated genes resulted in altered seed dormancy, allowing us to conclude that the decreased transcript levels of *GAI* and *HDA6* are likely to increase germination.

### GAI Regulation of Seed Dormancy

The DELLA *GAI* was the most AR-down-regulated gene in dry *sly1-2* seeds, suggesting a more important role in seed germination than previously believed. The DELLA *RGL2* is considered the major DELLA repressing seed germination, since *rgl2* mutations best rescue *ga1-3* germination in the light (Tyler et al., 2004; Cao et al., 2005). DELLA *GAI* also functions as a negative regulator of germination, since the *ga1-3 gai-t6 rgl2-1* triple but not the *ga1-3 rgl2-1* double mutant can germinate in the dark. DELLAs RGL2 and RGA mRNA and protein levels do not decrease with *sly1-2* after-ripening, whereas *GAI* mRNA levels decrease with dry after-ripening of *sly1* and Cvi (Supplementary Figure 13; Ariizumi and Steber, 2007). Mutant analysis confirmed that DELLA repressor *GAI* is a positive regulator of seed dormancy or a negative regulator of germination. Loss of function allele, *gai-t6*, increased germination, whereas gain-of-function allele *gai-1* promoted dormancy in the L*er* ecotype (**Figures 9A,B**). Moreover, the *gai-t6* mutation was able to partly rescue *sly1-2* germination without cold stratification, and strongly rescue *sly1-2* germination with cold stratification (**Figures 9C,D**). Thus, AR-down-regulation of *GAI* in dry *sly1-2* seeds likely results in increased germination potential since *GAI* acts as a positive regulator of *sly1-2* dormancy.

Previous work showed that *gai-1* has reduced germination potential compared to wild-type L*er* in cold-stratified seeds (Koornneef et al., 1985; Ariizumi et al., 2013). Moreover, *gai-t6* caused slightly increased germination without cold stratification, and slightly decreased germination with cold stratification of the low-dormancy ecotype Columbia-0 (Col) (Boccaccini et al., 2014). Thus, our model is that *GAI* transcript down-regulation with dry after-ripening increases germination potential by reducing GAI repressor levels during early imbibition. Further research will need to measure DELLA GAI protein levels during early seed imbibition.

### Control of Seed Dormancy by Histone Modification

Chromatin modifications regulate developmental processes including dormancy by altering gene transcription (reviewed in Nonogaki, 2014). Since 65% of the differentially-regulated transcripts in *sly1-2* (DvsWT) were down-regulated, it was interesting that rescue of *sly1-2* seed germination by long after-ripening was associated with down-regulation of the *HDA6* histone deacetylase because histone deacetylases repress gene transcription. Histone deacetylation represses gene expression through heterochromatin formation, whereas histone acetylation promotes gene expression and has been implicated in seed dormancy release by stimulating gene expression needed for seed germination. Our hypothesis was that *HDA6* down-regulation with after-ripening of *sly1-2* and Cvi breaks dormancy through increased expression of germination-promoting transcripts. The notion that *HDA6* stimulates seed dormancy was supported by the observation that loss of *HDA6* in the *sil1* mutant decreased seed dormancy in freshly harvested seeds (**Figure 10**). In addition to the *hda6/sil1* mutant, the histone deacetylase mutants *hda9* and *hda19* also exhibited reduced seed dormancy (Wang et al., 2013; van Zanten et al., 2014). *HDA9* is down-regulated with imbibition, but neither *HDA9* nor *HDA19* were down-regulated with *sly1-2* after-ripening. *HDA6* also appears to function in ABA and salt stress response, as *hda6* and *hda19* mutants were hypersensitive to ABA and salt inhibition of germination (Chen and Wu, 2010; Chen et al., 2010; Luo et al., 2012).

The increased seed dormancy associated with reduced GA signaling appears to be partially due to gene repression by histone deacetylation. The GA biosynthesis mutant *ga1-3* fails to germinate, and never regains the ability to germinate through after-ripening. Interestingly, the inhibitor of histone deacetylase activity TSA partly rescued the germination not only of *sly1-2* but of *ga1-3* seeds (**Figure 11**). The increased seed dormancy in *sly1-2* is rescued by long after-ripening, whereas the seed dormancy of the GA biosynthesis in *ga1-3* is not. No GA signaling can occur in *ga1-3*, whereas some GA signaling can occur in *sly1-2* mutants that cannot trigger DELLA destruction (Ariizumi and Steber, 2007; Ariizumi et al., 2013). Thus, DELLA-proteolysis independent GA signaling may be sufficient for *HDA6* down-regulation with *sly1* after-ripening. Taken together, this suggests that histone deacetylation maintains dormancy in GA mutants and that TSA-treatment may bypass GA signaling to relieve seed dormancy by allowing histone acetylation. This is consistent with previous studies suggesting that histone deacetylation stimulates and TSA relieves seed dormancy (Yoshida et al., 1995; Yano et al., 2013; van Zanten et al., 2014). Future work will need to examine whether down-regulation of *HDA6* with after-ripening is associated with altered histone acetylation of HDA6 targets.

### SLY1 and GA Signaling Regulate Protein Translation

Our *sly1-2* transcriptome studies indicate that regulation of translation-associated gene expression is one of the major roles of GA signaling in seeds (Nelson and Steber, 2017). Inhibitor studies showed that translation, not gene transcription, is required for seed germination *per se* (Rajjou et al., 2004). Thus, regulation of translation-associated genes is an excellent strategy for determining whether or not a seed can germinate. Consistent with this notion, previous studies found that translation-associated genes were strongly up-regulated with seed imbibition and Cvi after-ripening (Nakabayashi et al., 2005; Dekkers et al., 2016). Differentially regulated translation-associated genes in this and other studies included ribosomal subunits and translation initiation and elongation factors. The translation-associated category was strongly AR-up-regulated in imbibing L*er* wild-type seeds, but not well AR-up-regulated in imbibing *sly1-2* seeds (Dekkers et al., 2016; Nelson and Steber, 2017). The positive regulator of GA signaling, *SLY1*, was needed to up-regulate translation-associated genes with after-ripening of imbibed seeds (Nelson and Steber, 2017). Moreover, protein translation-associated transcripts were strongly GA-up-regulated and DELLA-down-regulated, indicating that regulation of translation-associated genes is a general function of GA signaling (Nelson and Steber, 2017). Previous work showed that after-ripening was associated with higher protein translation after 24h of imbibition in *H. annuus* (Layat et al., 2014). After-ripening can also be associated with increased translation of specific transcripts (Layat et al., 2014; Basbouss-Serhal et al., 2015). One possibility is that the increased mRNA accumulation of specific translation initiation factors with after-ripening is responsible for recruitment of specific transcripts. Future work will need to determine if dormant *ga1-3* and *sly1-2* seeds have either a general defect in protein translation or an inability to translate specific transcripts.

In contrast to imbibed seeds, translation-associated genes were strongly AR-down-regulated in dry *sly1-2* and Cvi seeds (**Figures 5B,C**). Although not as much as in DvsWT, translation-associated mRNAs accounted for 12% of the up-regulated transcripts in the *sly1-2* ARvsWT dry seed comparison (**Figure 5A**). This indicates that *SLY1* is not a requirement for this decrease with after-ripening, but that loss of *SLY1* resulted in a higher starting-point during seed maturation. Thus, it appears that *SLY1* is needed for down-regulation of translation-associated transcripts during seed maturation, since the translation-associated category accounted for 25% of the *sly1*-up-regulated genes in dry seeds (**Figure 5A**). This suggests that SLY1 may serve as a kind of shutdown signal to down-regulate translation associated genes during seed maturation to prepare for the quiescent state. In this context, it is interesting to note that *sly1-2* mutant seeds exhibit a mild decrease in survival of long-term storage (Ariizumi and Steber, 2007). Future work should examine the early imbibition proteome to determine if translation-associated proteins over-accumulate in *sly1-2* seeds during early imbibition. If too much of early translation is devoted to translation-associated gene expression, there may be limited amino acids available for protein synthesis of other important early-translated transcripts.

### Differences in mRNA Stability Correlate to Changes in Transcript Levels with Dry After-ripening

If changes in the dry seed transcriptome increase germination potential, then how can a quiescent, dry seed differentially regulate these changes in transcript levels? If we assume that *de novo* transcription is very unlikely in dry seeds, then such changes must be regulated through degradation that preferentially targets certain mRNAs over others. Genes that are up-regulated in transcriptome analyses may be those that are more stable or more well protected than the majority of the transcriptome, while those that are down-regulated are those that are less stable or otherwise more prone to degradation (i.e., targeted for degradation via mRNA oxidation or other mechanisms) than the majority. Consistent with this notion, comparison of dry seed AR-regulation with Arabidopsis mRNA stability, showed a correlation between AR-up-regulation and higher mRNA stability, as well as AR-down-regulation and lower mRNA stability (**Figure 7**). This is consistent with a previous study showing RNA degradation during dry after-ripening of sunflower seeds and Arabidopsis (Bazin et al., 2011; Basbouss-Serhal et al., 2017). Imbibed seeds did not show a correlation between mRNA stability and AR-regulation (Supplementary Figures 11A–C). In fact, in early Phase II (0h) there appeared to be a negative correlation between mRNA stability and AR-regulation, possibly indicating increased transcription of mRNAs that were not present in dry seeds at the time of imbibition due to lower stability.

Novel mechanisms may control those transcripts whose dry seed accumulation cannot be explained by differences in mRNA stability. Such genes may be regulated by other factors that increase or reduce the chances of degradation in a real seed. Future work should examine whether the subcellular localization of transcripts or RNA-binding proteins determine whether transcripts appear to be AR-up- or AR-down-regulated in dry seeds, as opposed to *de novo* transcription. Genes like *At3g23090* that have low stability mRNAs, but are up-regulated with after-ripening would be good candidates for such studies.

## Conclusion

How dormancy is lost in dry, metabolically inactive seeds is a fascinating question. This study took some first steps toward addressing this question by identifying transcriptional mechanisms underlying dormancy and dormancy loss in dry seeds of the GA-insensitive mutant, *sly1-2*. Our general model is that dry after-ripening of seeds leads to down-regulation of transcripts that negatively regulate seed germination. Loss of function mutations in two of these strongly AR-down-regulated transcripts, *GAI* and *HDA6*, resulted in increased germination potential (**Figures 9**, **10**). The AR-down-regulation of these two transcripts and of other transcription factors suggests that the control of gene transcription and of histone acetylation is one major mechanism controlling dormancy and after-ripening of dry seeds. The *sly1* seed dormancy phenotype was strongly associated with decreased abundance of transcription factor mRNAs, and generally skewed toward transcriptome down-regulation. Thus, it appears that over-accumulation of DELLA repressors has the general effect of down-regulating dry seed transcript abundances. There is one major counterexample to this observation; genes associated with protein translation were strongly up-regulated in dry dormant *sly1-2* seeds compared to wild type accounting for 25% of the *sly1*-up-regulated transcripts. Translation-associated genes are the major class of GA and *SLY1*-regulated transcripts in seeds (**Figures 5A,B**; Nelson and Steber, 2017). Ribosomes are inactive in dry seeds, and must be reactivated in order to germinate (Bewley et al., 2013). *SLY*1 is needed to down-regulate protein translation-genes during seed maturation and to up-regulate protein translation-genes with after-ripening during seed imbibition. Future work will need to examine if the increased dormancy of *sly1-2* and *ga1-3* results largely from inability to efficiently up-regulate protein translation.

## Author Contributions

CS provided the initial research design and obtained funding. TA performed the TSA experiments for **Figure 11**. SN performed all remaining experiments and bioinformatics analyses. Both CS and SN contributed to the research and analysis design, and to the writing of this article.

## Conflict of Interest Statement

The authors declare that the research was conducted in the absence of any commercial or financial relationships that could be construed as a potential conflict of interest.

## Footnotes

1 http://crb.wsu.edu/core-laboratories/molecular-biology-and-genomics-core

2 http://www.ebi.ac.uk/arrayexpress

3 http://arabidopsis.info/affy

4 www.virtualplant.org

5 https://github.com/bakuhatsu/microarrayTools

6 http://plntfdb.bio.uni-potsdam.de/v3.0/

7 http://arabidopsis.med.ohio-state.edu/AtTFDB/

8 http://planttfdb.cbi.pku.edu.cn

9 https://github.com/bakuhatsu/microarrayTools

10 http://structuralbiology.cau.edu.cn/PlantGSEA/

11 http://www.quantprime.de

12 www.virtualplant.org
