# Supplementary material for: Biology in the Dry Seed: Transcriptome Changes Associated with Dry Seed Dormancy and Dormancy Loss in the Arabidopsis GA-Insensitive sleepy1-2 Mutant
Source: Front Plant Sci. 2017 Dec 22;8:2158. doi: 10.3389/fpls.2017.02158 (PMC5744475; doi:10.3389/fpls.2017.02158)
Supplement: Supplementary file 3 [file Supplementary_Material.PDF]

Supplementary Material

Biology in the dry seed: transcriptome changes associated with dry seed dormancy and dormancy loss in the *Arabidopsis* GA-insensitive *sleepy1-2* mutant

Sven K. Nelson, Tohru Ariizumi, Camille M. Steber*

***Correspondence:** Camille M. Steber: csteber@wsu.edu

1. Supplementary Figures

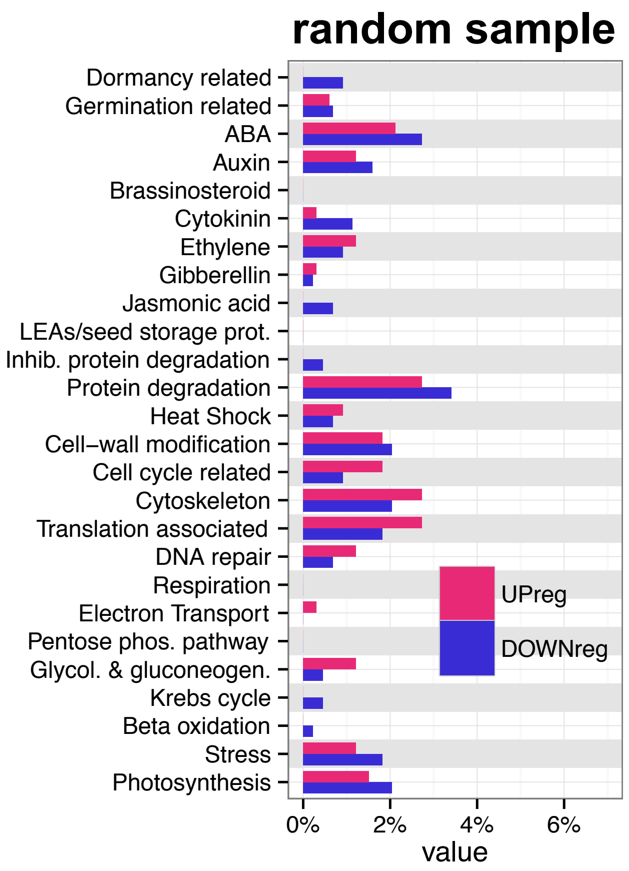

Supplementary Figure 1. TAGGIT ontology analysis of a randomly selected set of genes. Performed to determine if bias in the computational algorithm could cause similar enrichment of categories as was observed with after-ripening of *sly1-2* or Cvi by chance. For comparison to the *sly1-2* dataset, 330 up- and 430 down-regulated genes were selected at random (without overlap) and used for TAGGIT analysis.

```r
###############################
## Sven Nelson                ##
## 3/12/2015                  ##
## Function: countTFs         ##
###############################

countTFs <- function(geneListA, geneListB, A, B, labA, labB, title, latexTable, noPlot)
{
  # TFplot(TFcounts(DvsWTdryset$UP), TFcounts(DvsWTdryset$DN))
  TFplot(TFcounts(geneListA), TFcounts(geneListB),A, B, labA, labB, title, latexTable, noPlot)
}

TFcounts <- function(geneList) {
  # geneList is a list of AT numbers (not case sensitive)
  geneList <- toupper(geneList) # removes case-sensitivity
  ## Example code: UP and DOWN
  # ARvsD12h_UP_TFs <- TFcounts(ARvsD12hset$UP)
  # ARvsD12h_DN_TFs <- TFcounts(ARvsD12hset$DN)

  # This version takes a geneList of differentially expressed genes and returns
  # a dataframe with number of hits per TF type for easy plotting
  # Future versions may include the ability to return the list of genes in a given type

  # Please ensure that TFsWithType.tsv is in your working directory or TFsWithType exists
  if(is.null(TFsWithType)) {
    TFsWithType <- read.table("TFsWithType.tsv", header=TRUE, sep="\t")
  }
  if(is.null(TFsWithType)) {
    writeLines("\nUnable to locate 'TFsWithType' file.")
  }

  # Subset TFsWithType to a dataframe that only included genes in geneList with TF family
  TFhits <- subset(TFsWithType, Protein_ID %in% geneList)

  # Lists the different TF families present in geneList
  familiesRepresented <- unique(TFhits$Family)


  # Start by creating a data frame with 1 column for each TF family category
  TFfamilyCounts <- data.frame(matrix(NA, nrow = 1, ncol = length(familiesRepresented)))
  colnames(TFfamilyCounts) <- familiesRepresented

  # Fill in the dataframe with the number of hits in each TF family
  for(i in 1:length(TFfamilyCounts[1,])) {
    #TFfamilyCounts[,i] <- countHits(TFhits, familiesRepresented[i])

    TFfamilyCounts[,i] <- nrow(subset(TFhits, Family %in% familiesRepresented[i]))

    # Here is would be easy to modify this code to return a table of hits
    # subset(TFhits, familiesRepresented[i] %in% Family) # make use of this code
  }

  # returns a data.frame of counts for each TF family
  return(TFfamilyCounts)
}

### Takes two TFcounts objects and plots them using ggplot2
TFplot <- function(TFcountsA, TFcountsB, A = "UPreg", B = "DOWNreg", labA = A, labB = B, title = "TF families", latexTable=F, noPlot=F) {
  ## Example code: UP and DOWN
  # TFplot(ARvsD12h_UP_TFs, ARvsD12h_DN_TFs)
  ## Example code: diffA and diffB
  # TFplot(ARvsD12h_TFs, WTvsD12h_TFs,A="ARvsD12h",B="WTvsD12h")

  #### Preparing data ####
```

```
  # Use TFcounts to define TFcounts:
  # TFcountsA => UP, TFcountsB => DOWN

  # Make combined list of colnames (unique), ordered alphabetically

  combinedNames <- sort(unique(c(colnames(TFcountsA),colnames(TFcountsB)))) # combined list
  # Create a combined dataframe
  TFcluster.df <- data.frame(matrix(NA, nrow = length(combinedNames), ncol = 3))
  rownames(TFcluster.df) <- combinedNames
  colnames(TFcluster.df) <- c("Family","A","B")
  TFcluster.df$Family <- combinedNames

  # Fill in the dataframe with the number of hits in each TF family
  for(i in 1:length(combinedNames)) {
    #print(combinedNames)
    if (combinedNames[i] %in% colnames(TFcountsA)) {
      #print("enteredA")
      TFcluster.df$A[i] <- TFcountsA[1,combinedNames[i]]
    }
    if (combinedNames[i] %in% colnames(TFcountsB)) {
      #print("enteredB")
      TFcluster.df$B[i] <- TFcountsB[1,combinedNames[i]]
    }
  }
  #print(TFcluster.df)
  # Add code here to output a latex table (replace NAs with 0s)
  if(latexTable==TRUE) { # p-values get cut off, need to be rounded...
    TFclust <- TFcluster.df
    TFclust$A[is.na(TFclust$A)] <- 0
    TFclust$B[is.na(TFclust$B)] <- 0
    TFtable <- TFclust[,2:3]

    require(xtable)
    writeLines("\\documentclass[border={(0.5pt) (0.8pt) (1pt) (1pt)}]{standalone}

\\begin{document}
\\SweaveOpts{concordance=TRUE}\n")
    print.xtable(xtable(TFtable,digits = c(0,0,0),floating=FALSE))
    writeLines("\\end{document}")
    #print(tab,type="html")
  }

  #if (returnTable) {
  #  return(TFcluster.df)
  #} # nothing after this point will be run if table was returned
  if(!noPlot) {
    require(reshape) # for melt

    TFcluster.long <- melt(TFcluster.df,
                           ## ID variables:
                           # variables to keep but not split apart on
                           id.vars="Family",
                           # Measure variables: the source columns
                           measure.vars=c("B","A"),
                           # Name of the destination column that
                           # will identify the original
                           # column that the measurement came from
                           variable_name="Comparison"
    )

    # Reorder the data by Ontology
    TFcluster.long$Family <- factor(TFcluster.long$Family, levels = combinedNames)

    #require(scales) # For percent_format() in plot
    require(ggplot2)

    if(A == "UPreg" & B == "DOWNreg") { # UP => pinkish and DOWN => blueish
```

```
      colorA <- "#E82A76" # UP => pinkish
      colorB <- "#3B2DD6" # DOWN => blueish
    } else {
      colorA <- "#E69F00" # A => yellow
      colorB <- "#999999" # B => gray
    }

    #### Now for the TAGGIT plot ####
    ggplot(data=TFcluster.long, aes(x=Family, y=value, fill=factor(Comparison))) +
      geom_bar(position='dodge',stat='identity', width=0.8) +
      #scale_y_continuous(labels = percent_format()) +
      scale_y_continuous() +
      coord_flip() +
      xlab("") + # Set x-axis label
      ylab("Number of TFs") +
      theme_bw() +
      ggtitle(title) +
      scale_x_discrete(breaks=combinedNames, labels=combinedNames) +
      #theme(legend.position = c(.700, .250), legend.background = element_rect(fill = "transparent"), legend.text.align=0) +
      theme(legend.position = c(.800, .900), legend.background = element_rect(fill = "transparent"), legend.text.align=0) +
      labs(fill = NULL) +
      # colorA and colorB are reversed because this is a horizontal plot
      scale_fill_manual(breaks=c("A", "B"),values=c(colorB,colorA),labels = c(labA, labB))

    # For publications exported EPS at 400x511 resolution (??)
  }
}
```

Supplementary Figure 2. Code for the countTFs R function.

AGI locus	Gene	T$_a$	Direction	Name	Primer sequence (5'-3')	Reference
AT1G14920	*GAI*	70°C	forward	GAI-qRT1-F	CAATCAGTTCGCTATCGATTCG	Nomoto et al., 2012
			reverse	GAI-qRT1-R	CTTTCTGAACAGCTTCAGCGC	
AT5G63110	*HDA6*	70°C	forward	HDA6-qRT2-F	AACCTCGCATCTGGAGTGGAAC	current study
			reverse	HDA6-qRT2-R	ATCTTCACCGGTAGAGTCCCTGTC	
At5g45830	*DOG1*	52°C	forward	DOG1-qRT1-F	ATGGGATCTTCATCAAAGAAC	Mortensen et al., 2011
			reverse	DOG1-qRT1-R	CTTACGAAGCTTGTTATCATTATC	
At4g24210	*SLY1/sly1-2*	66°C	forward	SLY1-qRT3-F	TCTGTTGTGTTGGCGCTTGGTG	Chapter 2
			reverse	SLY1-qRT2-R	AGAGGCCAGAGGTAAAGAGAGTGG	
AT1G18100	*MFT*	66°C	forward	MFT-qRT2-F	AAACCCTCCACCGCAGTCAATC	current study
			reverse	MFT-qRT2-R	GGGAATATCCACGACAATCCAGTG	
At5g54070	*HSFA9*	68°C	forward	HSFA9-qRT1-F	AGACGGCAACGGAGACCGTCACCGTTGAAAGAG	Guan et al., 2013
			reverse	HSFA9-qRT1-R	TTGGGAAGTAGATTCTCTGAGAACTCGTAAGAATCC	
AT2G16060	*AHb1*	64°C	forward	AHb1-qRT1-F	AGAGACTTGGAGCCAGCCATTC	current study
			reverse	AHb1-qRT1-R	ACAATGCATACTTGGCCACCTC	
At2g17390	*ARK2B*	64°C	forward	ARK2B-qRT2-F	ACTCTGAAGGAAGGACTGCTTTGC	current study
			reverse	ARK2B-qRT2-R	AGAACCTGAGCACATCTCACCTC	

Supplementary Figure 3. Table of primers used for RT-qPCR with primer sequences and annealing temperatures used in this study.

GAI

dry	Ler (wt)	*sly1-2*(D)	*sly1-2*(AR)	*sly1-2 GID1b-OE*
Ler (wt)	–			
sly1-2(D)	0.0006	–		
sly1-2(AR)	0.0006	5.7E-06	–	
sly1-2 GID1b-OE	0.0006	*0.9882*	5.7E-06	–

HDA6

dry	Ler (wt)	*sly1-2*(D)	*sly1-2*(AR)	*sly1-2 GID1b-OE*
Ler (wt)	–			
sly1-2(D)	*0.3715*	–		
sly1-2(AR)	0.0003	0.0009	–	
sly1-2 GID1b-OE	*0.1651*	*0.4485*	0.0017	–

DOG1

dry	Ler (wt)	*sly1-2*(D)	*sly1-2*(AR)	*sly1-2 GID1b-OE*
Ler (wt)	–			
sly1-2(D)	*1.0000*	–		
sly1-2(AR)	0.0350	*0.0710*	–	
sly1-2 GID1b-OE	*0.7350*	*1.0000*	*0.1550*	–

SLY1

dry	Ler (wt)	*sly1-2*(D)	*sly1-2*(AR)	*sly1-2 GID1b-OE*
Ler (wt)	–			
sly1-2(D)	0.0010	–		
sly1-2(AR)	*1.0000*	0.0008	–	
sly1-2 GID1b-OE	0.0008	*1.0000*	0.0006	–

MFT

dry	Ler (wt)	*sly1-2*(D)	*sly1-2*(AR)	*sly1-2 GID1b-OE*
Ler (wt)	–			
sly1-2(D)	0.0001	–		
sly1-2(AR)	*1.0000*	0.0001	–	
sly1-2 GID1b-OE	0.0001	*1.0000*	0.0001	–

HSFA9

dry	Ler (wt)	*sly1-2*(D)	*sly1-2*(AR)	*sly1-2 GID1b-OE*
Ler (wt)	–			
sly1-2(D)	*0.0722*	–		
sly1-2(AR)	0.0002	0.0023	–	
sly1-2 GID1b-OE	*0.1887*	*0.4053*	0.0011	–

AHb1

dry	Ler (wt)	*sly1-2*(D)	*sly1-2*(AR)	*sly1-2 GID1b-OE*
Ler (wt)	–			
sly1-2(D)	*0.9874*	–		
sly1-2(AR)	0.0012	0.0008	–	
sly1-2 GID1b-OE	*0.9874*	*0.7143*	0.0022	–

Ler wt after-ripening time course

AHb1

dry	0wk AR (Ler wt)	1wk AR (Ler wt)	2wk AR (Ler wt)
0wk AR (Ler wt)	–		
1wk AR (Ler wt)	*0.2770*	–	
2wk AR (Ler wt)	0.0440	*0.2770*	–

Supplementary Figure 4. Tables of p-values for pairwise comparisons of RT-qPCR data. Significant values are indicated in black text. Significance is based on pairwise t-tests with Bonferroni-Holm correction for multiple comparisons with α = 0.07.

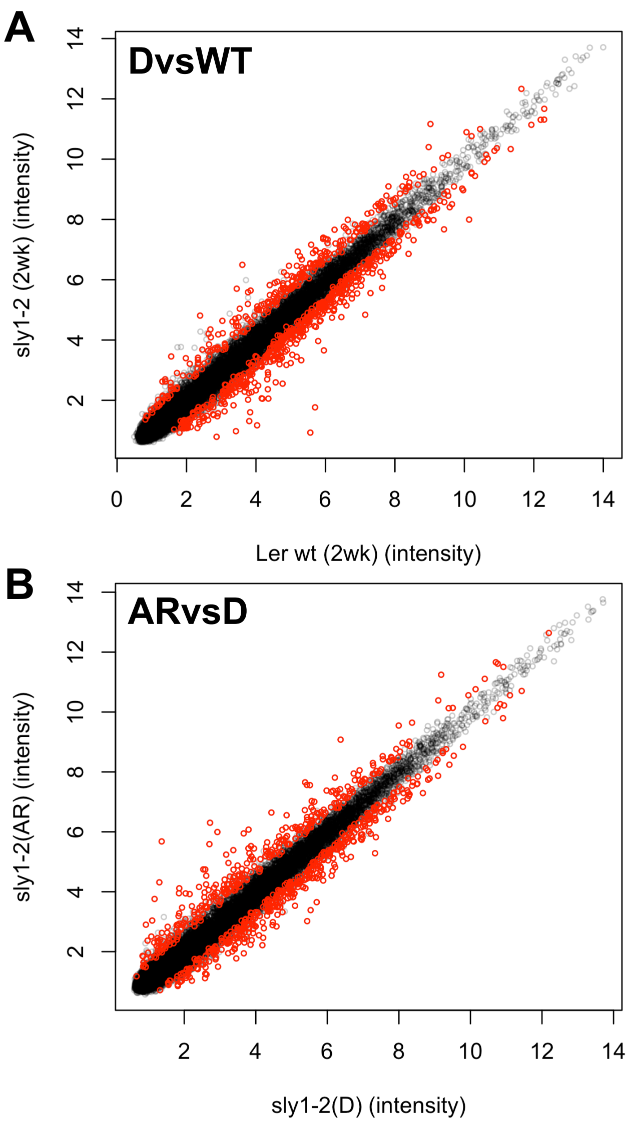

Supplementary Figure 5. Dry seed *sly1*-regulated and after-ripening regulated transcriptome differences. **(A)** Direct comparison of normalized intensities in *sly1-2*(D) and L*er* wt and **(B)** direct comparison of normalized intensities in *sly1-2*(AR) and *sly1-2*(D). In both comparisons differential regulation was observed at both high and low intensities.

A

sly1-regulated gene families

Gene family	Observed	Expected[a]	p-value
**UP-regulated**			
Cytoplasmic ribosomal protein gene family	54.1%	3.5%	5.21E-51
L23 ribosomal gene family	2.8%	0.0%	4.34E-03
**DOWN-regulated**			
Glycosyltransferase family 1	9.0%	1.6%	5.37E-04

[a]Expected indicates frequency of occurrence by chance

B

sly1-up-regulated GO enrichment

GO id	Term	Observed	Expected[a]	p-value
GO:0006412	translation	23.9%	1.8%	2.89E-46
GO:0034645	cellular macromolecule biosynthetic process	25.0%	5.8%	8.67E-22
GO:0009059	macromolecule biosynthetic process	25.0%	5.8%	8.69E-22
GO:0044267	cellular protein metabolic process	29.5%	8.2%	1.14E-21
GO:0019538	protein metabolic process	31.7%	10.6%	1.45E-18
GO:0022613	ribonucleoprotein complex biogenesis	7.5%	0.4%	5.23E-16
GO:0042254	ribosome biogenesis	7.5%	0.4%	5.23E-16
GO:0044249	cellular biosynthetic process	29.1%	10.4%	2.64E-15
GO:0009058	biosynthetic process	29.9%	11.1%	8.14E-15
GO:0044085	cellular component biogenesis	7.5%	0.7%	1.81E-12
GO:0071843	cellular component biogenesis at cellular level	7.5%	0.7%	1.81E-12
GO:0044260	cellular macromolecule metabolic process	32.8%	16.0%	6.10E-10
GO:0044238	primary metabolic process	39.9%	21.9%	1.29E-09
GO:0044237	cellular metabolic process	41.4%	24.0%	1.46E-08
GO:0043170	macromolecule metabolic process	33.6%	17.7%	1.85E-08
GO:0008152	metabolic process	49.3%	32.1%	1.90E-07
GO:0009987	cellular process	49.3%	34.0%	7.49E-06
GO:0071841	cellular component organization/biogenesis	9.0%	3.8%	3.91E-03

[a]Expected indicates frequency of occurrence by chance

C

sly1-down-regulated GO enrichment

GO id	Term	Observed	Expected[a]	p-value
GO:0042221	response to chemical stimulus	15.8%	7.6%	3.23E-06
GO:0050896	response to stimulus	25.1%	14.8%	3.23E-06
GO:0009628	response to abiotic stimulus	11.0%	5.4%	8.05E-04
GO:0010033	response to organic substance	9.7%	4.6%	8.28E-04
GO:0006979	response to oxidative stress	3.9%	1.1%	1.54E-03
GO:0010200	response to chitin	2.6%	0.5%	1.54E-03
GO:0009743	response to carbohydrate stimulus	3.2%	0.8%	1.70E-03
GO:0000103	sulfate assimilation	1.1%	0.1%	2.26E-03
GO:0006950	response to stress	14.3%	8.4%	2.69E-03
GO:0000302	response to reactive oxygen species	1.7%	0.3%	6.45E-03
GO:0009651	response to salt stress	4.3%	1.6%	8.05E-03
GO:0009737	response to abscisic acid stimulus	3.9%	1.4%	8.73E-03
GO:0019419	sulfate reduction	0.6%	0.0%	8.73E-03

[a]Expected indicates frequency of occurrence by chance

Supplementary Figure 6. Gene ontological analysis of transcriptome differences in the dry seed *sly1-2* DvsWT comparison. **(A)** Gene Family enrichment analysis using the BioMap online tool. **(B)** Biological Process Gene Ontology enrichment analysis of *sly1*-up-regulated genes and **(C)** of *sly1*-down-regulated genes.

A

AR-up-regulated GO enrichment

GO id	Term	Observed	Expected[a]	p-value
GO:0050896	response to stimulus	30.5%	14.8%	4.39E-09
GO:0009628	response to abiotic stimulus	14.8%	5.4%	1.21E-06
GO:0080167	response to karrikin	4.4%	0.5%	3.12E-06
GO:0042221	response to chemical stimulus	17.4%	7.6%	4.07E-06
GO:0006950	response to stress	18.5%	8.4%	6.21E-06
GO:0009611	response to wounding	4.0%	0.6%	4.86E-05
GO:0009737	response to abscisic acid stimulus	5.4%	1.4%	6.30E-04
GO:0009651	response to salt stress	5.4%	1.6%	3.41E-03
GO:0010033	response to organic substance	10.1%	4.6%	5.68E-03
GO:0006970	response to osmotic stress	5.4%	1.7%	5.92E-03

[a]Expected indicates frequency of occurrence by chance

B

AR-down-regulated GO enrichment

GO id	Term	Observed	Expected[a]	p-value
GO:0050896	response to stimulus	27.6%	14.8%	5.15E-08
GO:0006950	response to stress	17.3%	8.4%	5.87E-06
GO:0042221	response to chemical stimulus	16.0%	7.6%	5.87E-06
GO:0010035	response to inorganic substance	7.0%	2.0%	8.70E-06
GO:0009628	response to abiotic stimulus	12.3%	5.4%	3.46E-05
GO:0010038	response to metal ion	5.8%	1.7%	1.01E-04
GO:0046686	response to cadmium ion	5.0%	1.3%	1.21E-04
GO:0008152	metabolic process	43.6%	32.1%	1.46E-04
GO:0044237	cellular metabolic process	34.3%	24.0%	3.11E-04
GO:0044238	primary metabolic process	31.8%	21.9%	3.11E-04
GO:0044267	cellular protein metabolic process	15.0%	8.2%	4.01E-04
GO:0051716	cellular response to stimulus	7.8%	3.1%	4.01E-04
GO:0009987	cellular process	44.6%	34.0%	6.06E-04
GO:0006412	translation	5.5%	1.8%	6.52E-04
GO:0009617	response to bacterium	3.8%	1.0%	1.92E-03
GO:0033554	cellular response to stress	4.8%	1.6%	2.21E-03
GO:0042742	defense response to bacterium	3.3%	0.8%	2.21E-03
GO:0048583	regulation of response to stimulus	3.0%	0.7%	3.39E-03
GO:0080167	response to karrikin	2.5%	0.5%	3.39E-03
GO:0006970	response to osmotic stress	4.8%	1.7%	4.48E-03
GO:0009266	response to temperature stimulus	4.5%	1.6%	5.84E-03
GO:0009607	response to biotic stimulus	5.8%	2.4%	8.37E-03
GO:0044281	small molecule metabolic process	9.3%	4.9%	8.37E-03
GO:0048585	negative regulation of response to stimulus	1.8%	0.3%	8.37E-03

[a]Expected indicates frequency of occurrence by chance

Supplementary Figure 7. Biological Process Gene Ontology enrichment analysis of transcriptome differences in dry seeds with after-ripening. Analysis of **(A)** after-ripening-up-regulated and **(B)** after-ripening-down-regulated genes.

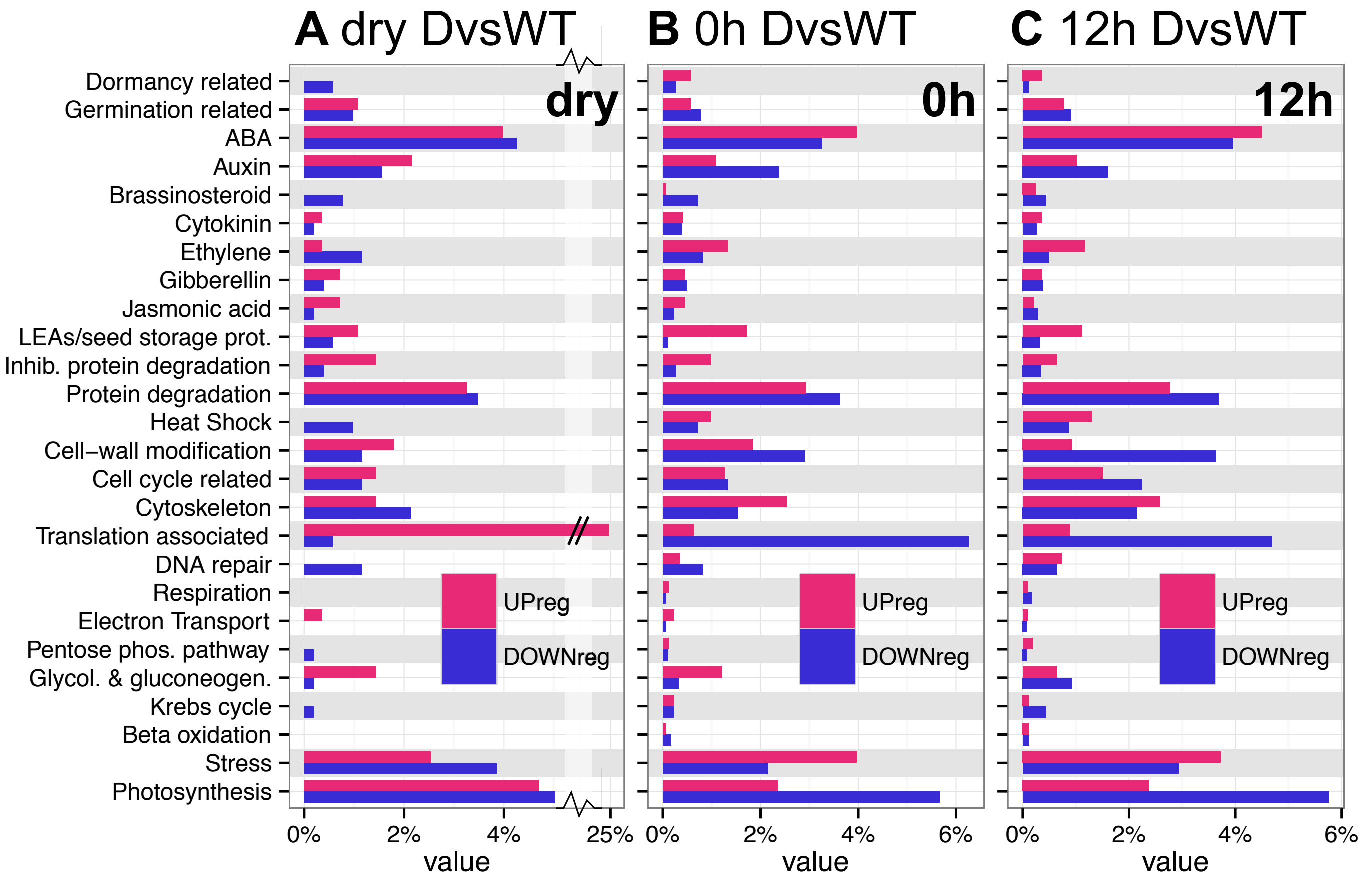

Supplementary Figure 8. TAGGIT gene ontology analysis of *sly1-2* DvsWT transcriptome changes. In **(A)**, dry seeds, **(B)**, at 0h in early Phase II, and **(C)**, at 12h in late Phase II . The value on the x-axis shows the percentage of either the total up-regulated or total down-regulated genes within a dataset. The 0h **(B)** and 12h **(C)** plots were originally published in Nelson and Steber, 2017 and are republished here for comparison with the dry seed timepoint.

A

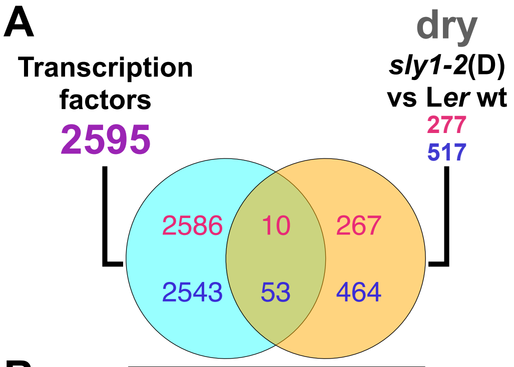

C

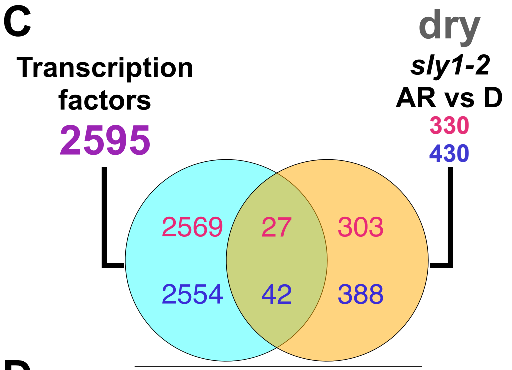

B

TF family	UP	DOWN
ABI3VP1	1	0
AP2-EREBP	0	5
ARF	0	1
bHLH	1	4
bZIP	0	3
C2C2-CO-like	0	1
C2H2	1	6
C3H	1	3
CAMTA	0	1
CCAAT	0	1
CSD	0	1
FAR1	0	2
GRAS	1	1
HB	1	1
HSF	0	1
LOB	0	1
MADS	1	0
MYB	0	2
MYB-related	0	4
NAC	0	3
Orphans	1	1
S1Fa-like	1	0
SET	0	1
SNF2	0	1
SOH1	0	1
TCP	0	1
Tify	0	1
TRAF	0	1
Trihelix	0	2
TUB	0	1
WRKY	1	2

D

TF Family	UP	DOWN
ABI3VP1	0	1
AP2-EREBP	3	7
ARF	0	3
AUX/IAA	1	0
BBR/BPC	0	1
bZIP	1	1
C2C2-GATA	1	0
C2H2	5	1
C3H	0	5
CAMTA	0	1
CCAAT	1	0
E2F-DP	0	1
EIL	1	0
G2-like	3	0
GRAS	0	4
GRF	0	1
HB	1	2
HSF	0	1
MADS	1	0
MBF1	2	0
MYB-related	0	2
NAC	1	1
Orphans	0	1
PHD	0	2
RWP-RK	0	3
S1Fa-like	1	1
SBP	0	1
SNF2	1	0
Tify	1	0
TRAF	0	1
WRKY	3	1

Supplementary Figure 9. Identification and categorization of transcription factors (TFs) differentially regulated in the *sly1-2* DvsWT comparison, and as a result of *sly1-2* after-ripening. **(A)** Venn diagram showing overlap of the list of 2595 TFs with the *sly1-2* DvsWT up- (red) and down-regulated (blue) fractions. **(B)** *sly1-2* DvsWT differentially regulated TFs categorized by TF family. **(C)** Overlap of the list of 2595 TFs with *sly1-2* after-ripening up- (red) and down-regulated (blue) fractions. **(D)** *sly1-2* after-ripening differentially regulated TFs categorized by TF family.

TF	Description		No. of genes	adj p-value[a]
**PIF1/PIL5**	bHLH, negatively regulates germination in dark	**All**	45	6.82×10^{-10}
		**Confirmed**	16	3.62×10^{-05}

[a]Significance by Fisher test with Yekutieli (FDR under dependency) ($p < 0.05$).

Supplementary Figure 10. Transcription factor target enrichment analysis of the dry seed DvsWT down-regulated dataset.

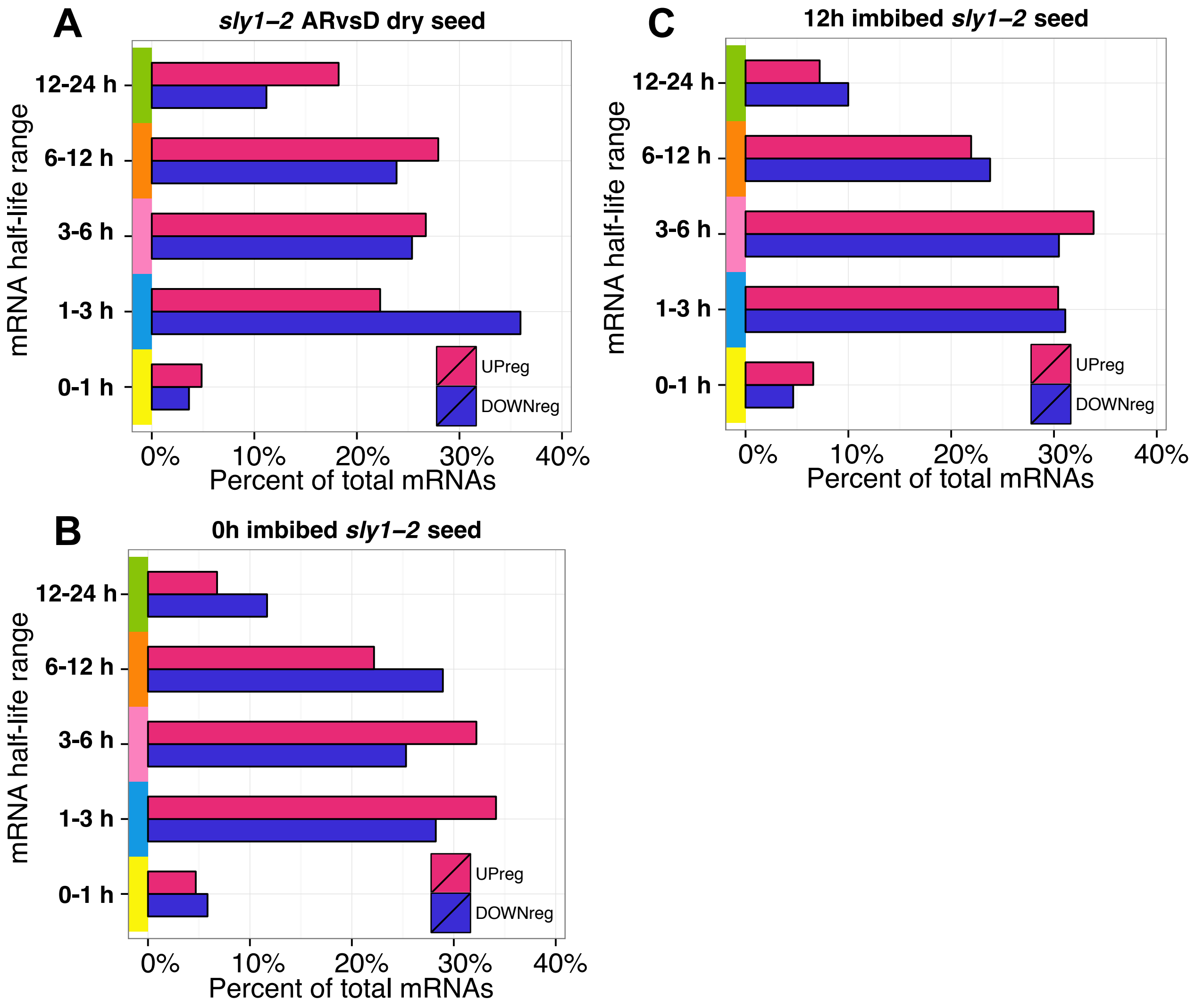

Supplementary Figure 11. Fractions of after-ripening-up- and down-regulated genes in each half life range stability category. **(A)** For *sly1-2* dry seed transcriptome changes, shown for comparison to imbibed seed data. **(B)** For *sly1-2* 0h imbibed seed transcriptome changes. **(C)** For *sly1-2* 12h imbibed seed transcriptome changes. The correlations between mRNA stability and up- or down-regulation with after-ripening were not apparent at 0h or 12h of imbibition.

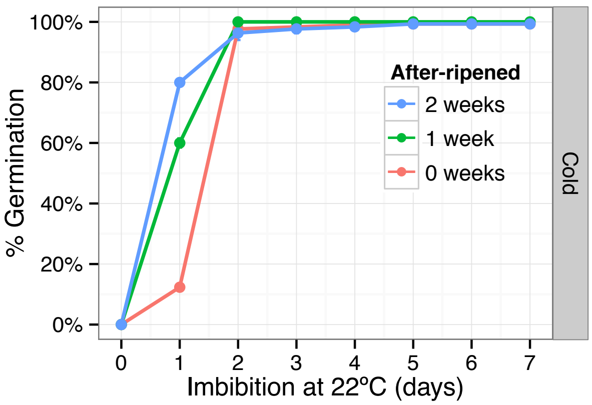

Supplementary Figure 12. Germination of L*er* wt used for RT-qPCR after-ripening time course. Seeds were imbibed on MS-agar plates and cold stratified for 4 d at 4°C, then moved to the light at 22°C where germination was scored.

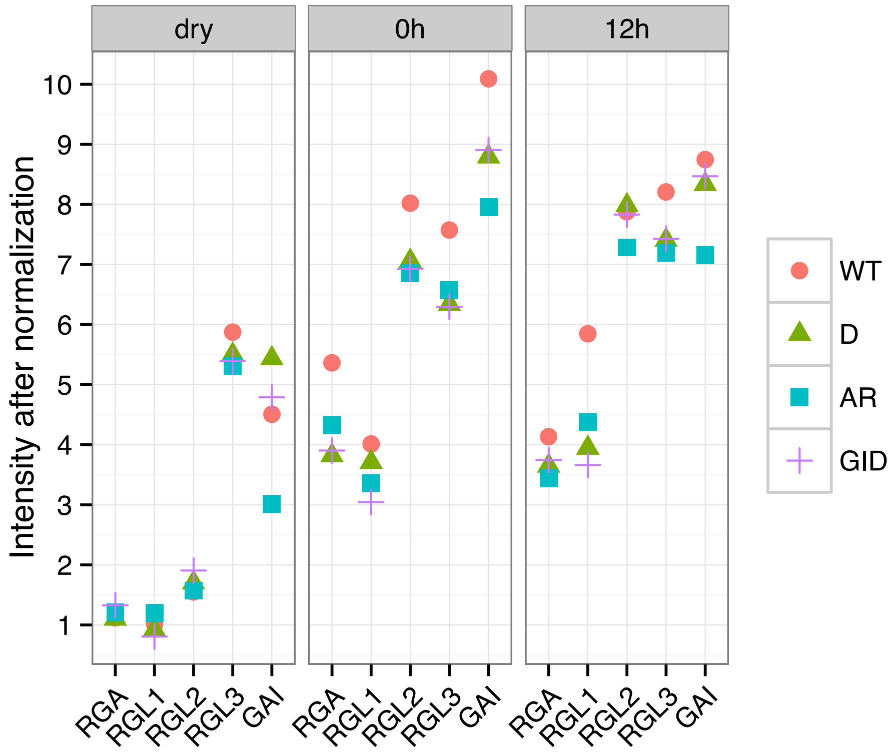

Supplementary Figure 13. Comparison of levels of DELLA gene transcripts at three stages of imbibition. Transcript level was estimated by plotting intensities after normalization for each of the five Arabidopsis DELLA genes. At the dry seed timepoint, only *GAI* and *RGL3* had high intensities, and only *GAI* showed after-ripening-dependent differential regulation. WT corresponds to L*er* wt, D corresponds to *sly1-2*(D), AR corresponds to *sly1-2*(AR), and GID corresponds to *sly1-2 GID1b-OE*.

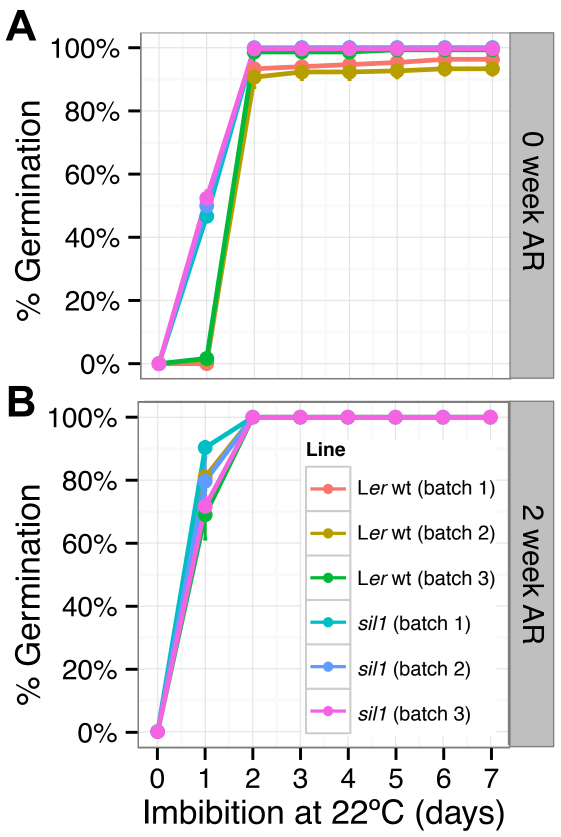

Supplementary Figure 14. A germination screen was performed to compare germination of *sil1* and L*er* wt harvested at near maturity with cold stratification. Seeds were germinated at two timepoints, **(A)** freshly harvested (0 week AR), and **(B)** 2 week old (2 week AR). "Cold" indicates that seeds were cold stratified for 4 d at 4°C, then moved to the light at 22°C where germination was scored daily. Three biologically independent batches of seed were assayed to clearly capture the *HDA6* loss of function phenotype in *sil1*.
